# Lowering Coordination Symmetry as a Strategy for Tuning Slow Magnetic Relaxation in Quasi-Isotropic Mn(II) Systems

**DOI:** 10.1021/acs.inorgchem.6c02639

**Published:** 2026-09-24

**Authors:** Jacek Sawka, Barbara Machura, Roman Boča, Cyril Rajnák, Maciej Witwicki, Dariusz Bieńko, Pavel Kopel, Alina Bieńko

**Affiliations:** † Faculty of Chemistry, University of Wroclaw, Joliot-Curie 14, 50-383 Wrocław, Poland; ‡ Institute of Chemistry, University of Silesia, Ninth Szkolna Street, 40-006 Katowice, Poland; § Faculty of Health Sciences, University of SS Cyril and Methodius, 91701 Trnava, Slovakia; ∥ Faculty of Natural Sciences, University of SS Cyril and, Methodius, 91701 Trnava, Slovakia; ⊥ Faculty of Chemistry, Wroclaw University of Science Technology, Wybrzeze Wyspianskiego 27, Wroclaw 50-370, Poland; # Department of Inorganic Chemistry, Faculty of Science, Palacky University, 17. listopadu 12, CZ-779 00 Olomouc, Czech Republic

## Abstract

Two pentacoordinate Mn­(II) complexes containing tridentate 2.2′-[oxybis­(methylene)] bis­(1*H*-1,3-benzimidazole) (OMeBB) ligand and pseudohalide coligands, [Mn­(OMeBB)­(NCS)_2_] (**1**) and [Mn­(OMeBB)­(NCO)_2_] (**2**), exhibit a very rare {Mn^II^N_4_O} coordination mode involving semicoordinate bonds between the ether oxygen atom of the ligand and the Mn­(II) center. Susceptibility, magnetization, EPR and *ab initio* calculations confirm only small magnetic anisotropy. The AC susceptibility measurements reveal field-induced slow magnetic relaxation over the broadest temperature range reported so far for Mn­(II) systems, extending up to *T* ≈ 30 K, with two or three relaxation channels predominantly governed by the direct relaxation mechanism. These results demonstrate that low coordination symmetry and unconventional ligand topology provide an effective approach for tuning magnetic relaxation behavior in quasi-isotropic Mn­(II) systems.

## Graphical abstract

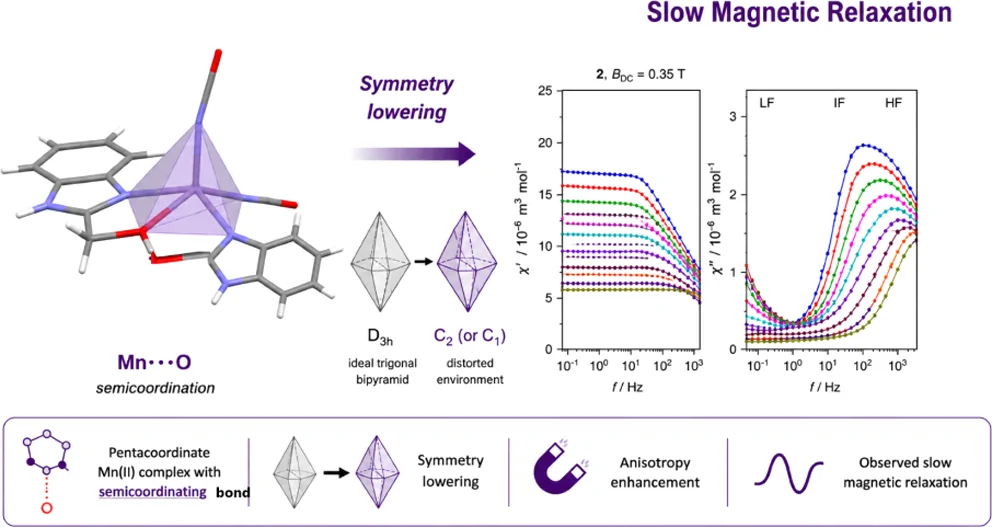

## Introduction

The design of transition-metal complexes exhibiting slow magnetic relaxation remains an important challenge in molecular magnetism, particularly for systems with intrinsically weak magnetic anisotropy.^1^ The axial zero-field splitting parameter *(D*) is commonly regarded as a key factor governing slow magnetic relaxation (SMR) because it determines both the magnetic anisotropy and the effective energy barrier for magnetization reversal.^2,3^ This paradigm has been particularly successful in lanthanide-based systems, where large anisotropy leads to well-defined Orbach-type relaxation processes.^4,5^ However, recent studies have demonstrated that large *D* values are not a strict prerequisite for the observation of slow magnetic relaxation, particularly in 3d transition-metal systems, where spin dynamics may instead be governed by alternative relaxation mechanisms involving spin–phonon coupling, quantum tunneling, or direct relaxation processes. Among 3d metal ions, high-spin Mn­(II) (3d,^5^ *S* = 5/2) represents one of the most demanding cases because its orbitally nondegenerate ^6^A_1_ ground state results in nearly isotropic magnetic behavior and very small zero-field splitting. Consequently, Mn­(II) complexes were long considered poor candidates for slow magnetic relaxation phenomena, since the *D* parameter in most Mn­(II) compounds usually does not exceed 0.5 cm^–1^.^6^ Nevertheless, several examples of field-induced slow magnetic relaxation have been reported for Mn­(II) systems^7–18^ with diverse coordination numbers and donor environments, while emphasizing that mononuclear Mn­(II) examples remain scarce.

In these compounds, the magnetic dynamics are not governed by large anisotropy barriers but rather by subtle structural distortions, low local symmetry, intermolecular interactions, and spin–phonon coupling effects. An early example was reported by Rajnák et al.,^14^ who demonstrated field-induced slow magnetic relaxation in a mononuclear Mn­(II) complex with very small magnetic anisotropy, highlighting that alternative relaxation mechanisms may dominate in quasi-isotropic Mn­(II) systems. These findings clearly indicate that although large anisotropy is not strictly required, a finite degree of anisotropy remains necessary to induce slow magnetic relaxation, making the structural origin of weak anisotropy a key issue in the design of Mn­(II)-based molecular magnetic materials. In this context, low-coordinate Mn­(II) systems provide particularly attractive platforms for investigating how subtle structural distortions, coordination symmetry, and spin–phonon interactions influence spin dynamics in quasi-isotropic systems. A promising strategy for inducing slow magnetic dynamics involves the deliberate design of low-symmetry coordination environments capable of generating anisotropic ligand fields despite the electronic isotropy of the d^5^ metal center. In particular, five-coordinate Mn­(II) complexes are of special interest because geometrical distortion may induce measurable magnetic anisotropy and untypical relaxation pathways. However, such systems remain extremely rare.^18^

Benzimidazole-based ligands are especially suitable for stabilizing low-coordinate metal centers because of their flexible coordination behavior and their ability to generate structurally constrained environments. Furthermore, pseudohalide ligands such as thiocyanate and cyanate can effectively modulate both local geometry and intermolecular interactions, providing an opportunity to investigate structure–property relationships governing magnetic relaxation behavior.

Along this line, we report two mononuclear pentacoordinate Mn­(II) complexes, [Mn­(OMeBB)­(NCS)_2_] (**1**) and [Mn­(OMeBB)­(NCO)_2_] (**2**), featuring a rare {Mn^II^N_4_O} coordination environment and distorted square-pyramidal geometry. Magnetic measurements, EPR spectroscopy and *ab initio* calculations reveal only small magnetic anisotropy; nevertheless, both compounds exhibit field-induced slow magnetic relaxation with multiple relaxation channels observable up to ca. 30 K. Importantly, the relaxation dynamics are governed by direct and phonon-mediated mechanisms rather than an Orbach process. To the best of our knowledge, this is the first example of slow magnetic relaxation observed for Mn­(II) complexes featuring this unusual N_4_O coordination mode. These results demonstrate that low coordination symmetry and unconventional ligand topology provide an effective approach for tuning magnetic relaxation behavior in quasi-isotropic Mn­(II) systems.

## Experimental Section

### Synthetic Procedures

The air-stable manganese­(II) complexes [Mn­(OMeBB)­(NCS)_2_] (**1**) and [Mn­(OMeBB)­(NCO)_2_] (**2**), were obtained as light brown crystals (for **(1)**) and cream colors (for **(2)**) successively by reacting methanol solution of 2.2’-[oxybis­(methylene)]­bis­(1*H*-1,3-benzimidazole) (OMeBB) with MnCl_2_·4H_2_O and suitable pseudohalide ligand: KSCN (for **(1)**) and NaNCO (for **(2)**) at room temperature. Details of synthesis and analysis are described in Supporting Information.

### IR Studies

Elemental analysis was registered by Vario EL III apparatus (Elementar, Germany).

IR spectra were recorded on a Brucker spectrophotometer in the spectral range 4000–40 cm^–1^ with the samples as of KBr pellets (Figures S1, S2 ESI†).

### Crystal Structure Determination and Refinement

To confirm the phase purity of the synthesized Mn­(II) complexes, the XRPD spectra of these compounds were recorded. Powder X-ray diffraction (XRPD) measurements on **(1)** and **(2)** were performed on a PANalytical Empyrean X-ray diffractometer by using Cu–Kα radiation (λ = 1.5418 Å), in which the X-ray tube was operated at 40 kV and 30 mA ranging from 5° to 50° (Figures S3 and S4). As shown in Figures S3 and S4, the XRPD patterns measured for polycrystalline samples **(1)** and **(2)** were in good agreement with the XRPD patterns simulated from the respective single-crystal X-ray data using the Mercury program,^19^ demonstrating that the crystal structures are truly representative of the bulk materials.

Single crystal X-ray diffraction data was collected at room temperature with Oxford Diffraction Gemini A Ultra diffractometer, equipped with CCD Atlas detector and graphite monochromated MoKα (λ = 0.71073 Å) radiation source. Data collection, cell refinement and data reduction were performed with CrysAlisPro software, and the structures of complexes **(1)** and **(2)** were solved and refined with Olex2 1.5 software, with SHELXS-2014 and SHELXL-2014 package.^20–22^ All non-hydrogen atoms were refined with anisotropic thermal parameters by full-matrix least-squares on F^2^, while the hydrogen atoms were calculated and refined using the riding-model idealized positions, with fixed isotropic displacement parameters: U_iso_(H) = 1.2, U_eq_(C), d­(C–H) = 0.93 Å for aromatic hydrogens; U_iso_(H) = 1.2, U_eq_(C), d­(C–H) = 0.97 Å for CH_2_; U_iso_(H) = 1.2 U_eq_(N), d­(N–H) = 0.86 Å for imidazole N–H. Data visualization and analyses were performed using Mercury 2025.2.0 2, PLATON^23^ and SHAPE 2.0 ^24^ software. The X-ray structures were deposited at CCDC numbers: 2551333 **(1)** and 2551334 **(2)** and they can be obtained free of charge from The Cambridge Crystallographic Data Center at www.ccdc.cam.ac.uk/getstructures.

### Electron Paramagnetic Resonance

Electron paramagnetic resonance (EPR) measurements were carried out using a Bruker EMX Plus cw-EPR spectrometer operating at 9.7 GHz (X-band). The spectrometer was equipped with a teslameter and a frequency counter. All spectra were recorded at 80 K for carefully ground samples and simulated using EasySpin.^25,26^ All the EPR parameters were obtained using the spin Hamiltonian

1 $$Ĥ=\mu_{B}\mathrm{B⃗}\cdot \overset{®}{\overset{®}{g}}\cdot \mathrm{S⃗}+D\left[{Ŝ}_{z}^{2}-S\left(S+1\right)/3\right]+E\left({Ŝ}_{x}^{2}-{Ŝ}_{y}^{2}\right)$$

by optimizing the agreement between the simulated and experimental spectra.

### Theoretical Calculation

Quantum chemical calculations were performed using the ORCA software package.^27–29^ The calculations were performed using the ZORA Hamiltonian with the ZORA-def2-SVP basis set and the RIJCOSX approximation together with TightSCF convergence criteria and DEFGRID2 integration grids. For Mn and N atoms, the ZORA-def2-TZVPP basis set was applied. The CASSCF calculations were performed with an active space of 5 electrons in 5 orbitals. The orbital optimization employed the KDIIS SwitchStep procedure. Calculations were carried out for spin multiplicities of 6, 4, and 2, considering 1, 24, and 75 roots, respectively. The RI TrafoStep approach was used together with relativistic treatment, while spin–orbit coupling effects were included.

### Magnetic Data

The DC magnetic data was taken using the SQUID magnetometer (MPMS, Quantum Design) with ca. 25.42 **(1)** or 27.14 **(2)** mg of sample. The susceptibility data was acquired at the *B*_DC_ = 0.5 T between *T* = 1.8 and 300 K. This was corrected to the underlying diamagnetism and transformed to the effective magnetic moment. The magnetization data was taken at low temperatures *T* = 2.0 up to *B* = 5.0 T. No remnant magnetization has been detected.

The AC susceptibility data was taken with the same apparatus and the same samples using the amplitude of the oscillating field *B*_AC_ = 0.3 mT. Two regimes were used: (i) scan of the AC susceptibility as a function of the field up to *-B*_DC_ = 1 T at *T* = 2.0 K for a set of four trial frequencies f; (ii) scan of the AC susceptibility as a function of temperature for 22 frequencies of the oscillating field and a set of temperatures ranging between *T* = 1.8 and 7.0 (8.0) K, all performed at the properly selected field *B*_DC_ = 0.3 T for complex **(1)** and 0.35 T for **(2)**. The magnetic data was analyzed by own software.^30^

## Results and Discussion

### Structural Description

The complexes [Mn­(OMeBB)­(NCS)_2_] (**1**) and [Mn­(OMeBB)­(NCO)_2_] (**2**) crystalize in the centrosymmetric triclinic (*P*1̅) and monoclinic (*C*2/*c*) space groups, respectively (Table S1). In both molecules, the central Mn­(II) ion is five-coordinated by two N-bonded pseudohalide ions (NCS^–^ in **(1)** and NCO^–^ in **(2)**) and by the tridentate ligand L 2.2’-[oxybis­(methylene)]­bis­(1*H*-1,3-benzimidazole), coordinated through two nitrogen and one oxygen donor atoms (Figure 1).

**Figure 1:**
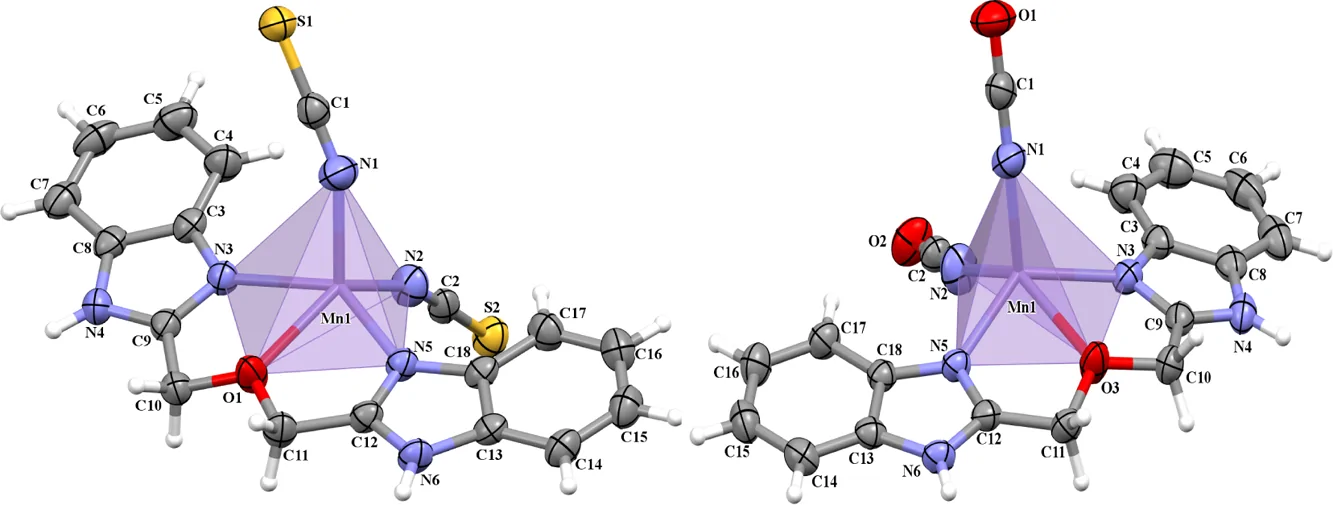
Molecular structures **(1)** and **(2)** with the atom numbering. Thermal ellipsoids of non-hydrogen atoms are given at 50% probability level. CCDC numbers: 2551333 (**1**) and 2551334 (**2**).

Upon coordination to Mn­(II), 2.2′-[oxybis­(methylene)]­bis­(1*H*-1,3-benzimidazole) forms two five-member chelate rings with the bite angles 68.64(6) and 69.35(6)° for **(1)**, and 69.00(5) and 69.03(5)° for **(2)**. The M–N_L_ bond lengths are elongated by 0.065–0.152 Å relative to those between the metal ion and the quasi-linear pseudohalides anions (Table S2), indicating the stronger coordination of the latter ones, as expected for such systems.^31^ Compared to the Mn–N_L_ distances [2.0445(18)–2.1971(16) Å], the Mn–O_L_ bond lengths are noticeably longer [2.3817(16) Å for **(1)** and 2.3489(14) Å for **(2)**]. The elongation of the Mn–O_L_ bond lengths appears to be a characteristic structural feature of 2.2′-[oxybis­(methylene)]­bis­(1*H*-1,3-benzimidazole), which can coordinate in both bidentate and tridentate modes.^31^ The elongated Mn–O_L_ distances are considered as semicoordinate bonds, corresponding to an intermediate bonding regime between conventional coordination bonds and nonbonding interactions.^32,33^ Replacement of NCS^–^ (in **(1)**) by NCO^–^ (in **(2)**) induces minor variations in bond length values, generally leading to a slight shortening of Mn–N_NCX_ and Mn–O_L_, accompanied by a small elongation of Mn–N_L_ distances (Table S2). More significant changes are observed in the bond angles upon the replacement of NCS^–^ (**1)** by NCO^–^ (**2**). In particular, a larger bending in the M–N–C­(–S) linkage is observed in complex **(1)**, while the dihedral angle between the benzimidazoles planes of the ligand L are approximately two times smaller in **(1)** (7.68°) relative to **(2)** (16.50°), leading to the enlargement of N(3)–Mn(1)–N(5), 136.80(7)° for **(1)** and 132.23(6) ° for **(2)**.

In agreement with the angular structural index parameter τ_*5*_ ^34^ defined as the difference between the two largest angles divided by 60, and calculated as 0.002 for **(1)** and 0.150 for **(2)**, the geometries of both complexes are best described as a distorted square-pyramidal.

Further confirmation of the square-pyramidal coordination environment around the Mn­(II) center in both structures has been provided by the continuous symmetry measures (CSM) analysis,^20^ in which the deviation from an ideal reference polyhedron is quantified by the shape measure *S*_*Q*_ *(P)* (Table S3). The obtained (*S*_*Q*_ *(P)*) values relative to the square-pyramid geometry, 2.922 for **(1)** and 1.730 for **(2)**, are markedly smaller than the corresponding distances to the ideal trigonal bipyramid, 5.119 for **(1)** and 6.489 for **(2)**. The displacement of the manganese­(II) ion from the least-squares basal plane defined by three donor atoms of L and N(1) atom of the pseudohalide ion are 0.636 Å for **(1)** and 0.682 Å for **(2)** toward the apical position.

A search in Cambridge Structural Database (CSD, version 2025.2.0) revealed that the most common coordination number among thiocyanate and cyanate manganese­(II) complexes is six. Five-coordinate pseudohalide manganese­(II) coordination compounds are exceedingly rare, with only nine such structures reported to date. As demonstrated in Table S4, the geometry around the Mn­(II) center in these compounds varies between distorted square-pyramidal and trigonal-bipyramidal arrangements, depending on the nature of the coordinated ligands. Based on these findings, it can be assumed that tridentate ligands incorporating imidazole moieties tend to favor the formation of complexes with a geometries close to a square pyramid (Table S4).^35,36^ The present systems therefore represent uncommon five-coordinate pseudohalide manganese­(II) complexes stabilized by the tridentate benzimidazole-based ligand. Furthermore, the donor set and OMeBB ligand topology make **1** and **2** rare examples among five coordinate Mn­(II) pseudohalide structures. Both obtained compounds feature a unique {Mn^II^N_4_O} coordination environment with a semicoordinate Mn–O bond and minor angular deviations from the ideal square-pyramidal geometry. Notably, compound **1** exhibits the lowest τ_*5*_ parameter among five-coordinate pseudohalide manganese­(II) complexes reported to date (Table S4).

A summary of the crystal packing analysis for complexes **(1)** and **(2)**, performed with the use of Mercury 2025.2.0^19^ and PLATON^23^ software, is provided in the Tables S5–S7 and Figures S5–S8. In both crystal structures, the pyrrole-type hydrogens (N–H) of the benzimidazole rings participate in hydrogen bond formation with sulfur **(1)** and oxygen **(2)** atoms of the pseudohalide ions (Table S5 and Figures S5–S6). Besides the N–H···X contacts (X=S in **(1)** and O in **(2)**), also π···π stacking interactions between planar benzimidazole rings are crucial for the supramolecular assembly of [Mn­(L)­(NCX)_2_] molecules in the solid state. Furthermore, the crystal structure of **(2)** is stabilized by C–H···π contacts (Tables S4–S6 and Figures S5–S8).

### EPR Spectra

The X-band EPR spectra of crystalline powder samples of **(1)** and **(2)** exhibit very broad signals (Figure 2). This significant line broadening arises from intermolecular dipole–dipole interactions and *D*-strain and prevents the resolution of hyperfine splitting due to ^55^Mn nuclei. Nevertheless, features attributable to ZFS are partially resolved, indicating that the ZFS is sufficiently large to influence the X-band EPR spectra of both compounds.^6,37,38^ The spin Hamiltonian parameters {*D*, *E*, *g*_*x*_, *g*_*y*_, *g*_*z*_} were estimated through spectral simulations.^25,26^ It should be noted that, due to magnetic field limitations, the full spectrum of compound **(2)** could not be recorded. As expected for Mn­(II) ions, the anisotropy of the Zeeman interaction is negligible and the *g*-values are close to the free electron value, with *g*_*i*_ = 2.01 for **(1)** and *g*_*i*_ = 2.00 for **(2)**. The ZFS parameters were determined as |*D*| = 0.06 cm^–1^ and |*E*/*D*| = 0.12 for **(1)** and |*D*| = 0.14 cm^–1^ and |*E*/*D*| = 0.10 for **(2)**. However, it should be acknowledged that the simulations for **(1)** exhibit a modest sensitivity to the rhombicity parameter |*E*/*D*|. Comparable agreement with the experimental spectrum is obtained for the |*E*/*D*| values between approximately 0.05 and 0.15, indicating that this parameter cannot be determined with high precision. In contrast, the simulations of **2** are considerably more sensitive to |*E*/*D*|. The best agreement is obtained for |*E/D*| = 0.10. Decreasing this value leads to a poorer reproduction of the low-field region of the spectrum, whereas increasing it deteriorates the agreement in the high-field region. The dependence of the simulated spectra on |*E*/*D*| and |*D*| is shown in Figures S10 and S11, respectively. The |*E*/*D*| ratios indicate a significant rhombicity in ZFS (over 30% of the maximum possible value of 1/3). Comparable |*E*/*D*| values have been reported for other Mn­(II) complexes.^38–43^

**Figure 2:**
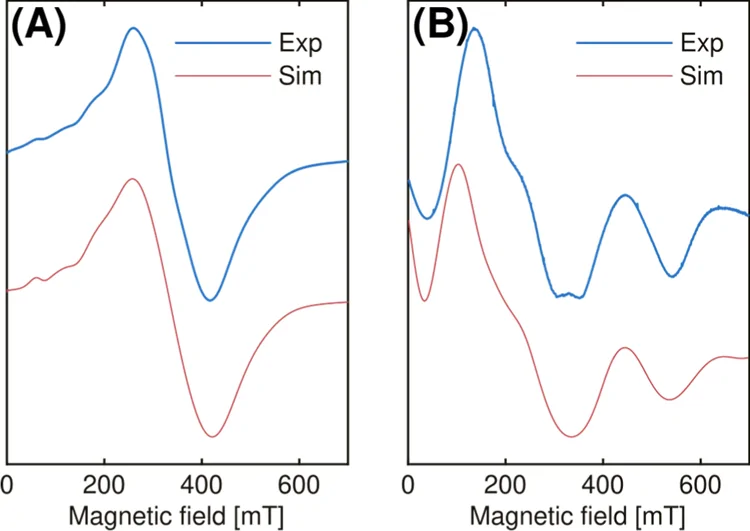
X-band EPR spectra of powdered **(1)** (A) and **(2)** (B).

Regarding the origin of the differences in the ZFS parameters between **(1)** and **(2)**, they exhibit coordination geometries that are close to distorted square-pyramidal and the observed structural differences are relatively small. Therefore, the differences in the ZFS parameters are unlikely to arise solely from the different degrees of distortion from the ideal square-pyramidal geometry. Instead, replacement of NCS^–^ by NCO^–^ modifies the ligand field through changes in the ligand donor properties and metal–ligand covalency. In addition, replacement of the oxygen atom in NCO^–^ by the heavier sulfur atom in NCS^–^ may increase ligand-mediated spin–orbit coupling contributions to ZFS. The influence of heavier atoms on spin–orbit interaction has been showed in transition-metal complexes, including the effect on ZFS in Mn­(II).^44–46^ Interestingly, despite the heavier sulfur atom, complex **(1)** exhibits a smaller experimental |*D*| value than **(2)**. This observation suggests that the additional spin–orbit coupling contribution in **(1)** associated with the NCS^–^ ligand does not simply increase the magnitude of the ZFS but may instead have an opposite sign. Such an interpretation is qualitatively in line with the CASSCF/NEVPT2 calculations, which predicted a negative D value for **(1)** and a positive D value for **(2)**.

The results of the *ab initio* calculations performed for the experimental geometry of **(1)** and **(2)** are presented in Table 1 and Table S8. They confirm all g-tensor components *g*_*i*_ = 2.002 and a very small ZFS parameter *D*/*hc* = −0.05 and +0.07 cm^–1^. Overall, these values are consistent with those obtained from the EPR measurements.^47^ The agreement between the CASSCF/NEVPT2 and experimentally derived ZFS parameters is sometimes semiquantitative. Such discrepancies have been reported for Mn­(II) systems and arise from the high sensitivity of the ZFS to subtle changes in the electronic structure, including metal–ligand covalency, the description of excited states (sextet-quartet excitation energies in particular), dynamic correlation effects and the molecular geometry.^6,37,48^ Furthermore, the calculated parameters correspond to an isolated molecule. Also, the experimental determination of the rhombicity parameter for **(1)** has some uncertainty, as discussed above, complicating a direct comparison of these theoretical and experimental values.

**Table 1:** Magnetic Parameters From ab Initio Calculations, DC Magnetic Measurements and X-band EPR Spectroscopy

parameter	1 (-NCS)	2 (-NCO)
***Ab initio calculations***		
All *g*	2.002	2.002
*D*/cm^–1^	–0.054	+0.075
*E*/|*D*|	0.270	0.040
***EPR measurements***		
All *g*	2.01	2.00
|*D*|/cm^–1^	0.06	0.14
*E*/cm^–1^	0.0072	0.014
*|E*/*D*|	0.12	0.10
***DC magnetic data***		
*g*_iso_	2.00(5)	1.99(1)
*D*/cm^–1^	0.41(27)	0.48(7)
χ_TIM_/10^–9^ m^3^ mol^–1^	–10	–9.8

### DC Magnetic Data

The DC magnetic data for both complexes are displayed in Figure 3. As shown, the magnetic susceptibility of **(1)** increases monotonically upon cooling. The calculated effective magnetic moment remains nearly constant down to low temperature, consistent with an effective spin *S* = 5/2. At the lowest temperature range, a slight decrease in the effective magnetic moment is observed, indicating small zero-field splitting (ZFS). The magnetization per formula unit for **(1)** approaches *M*_mol_/*N*_A_ = 5 μ_B_, further confirming the *S* = 5/2 state and eventually small ZFS. The magnetic behavior of **(2)** is very similar to that of **(1)**.

**Figure 3:**
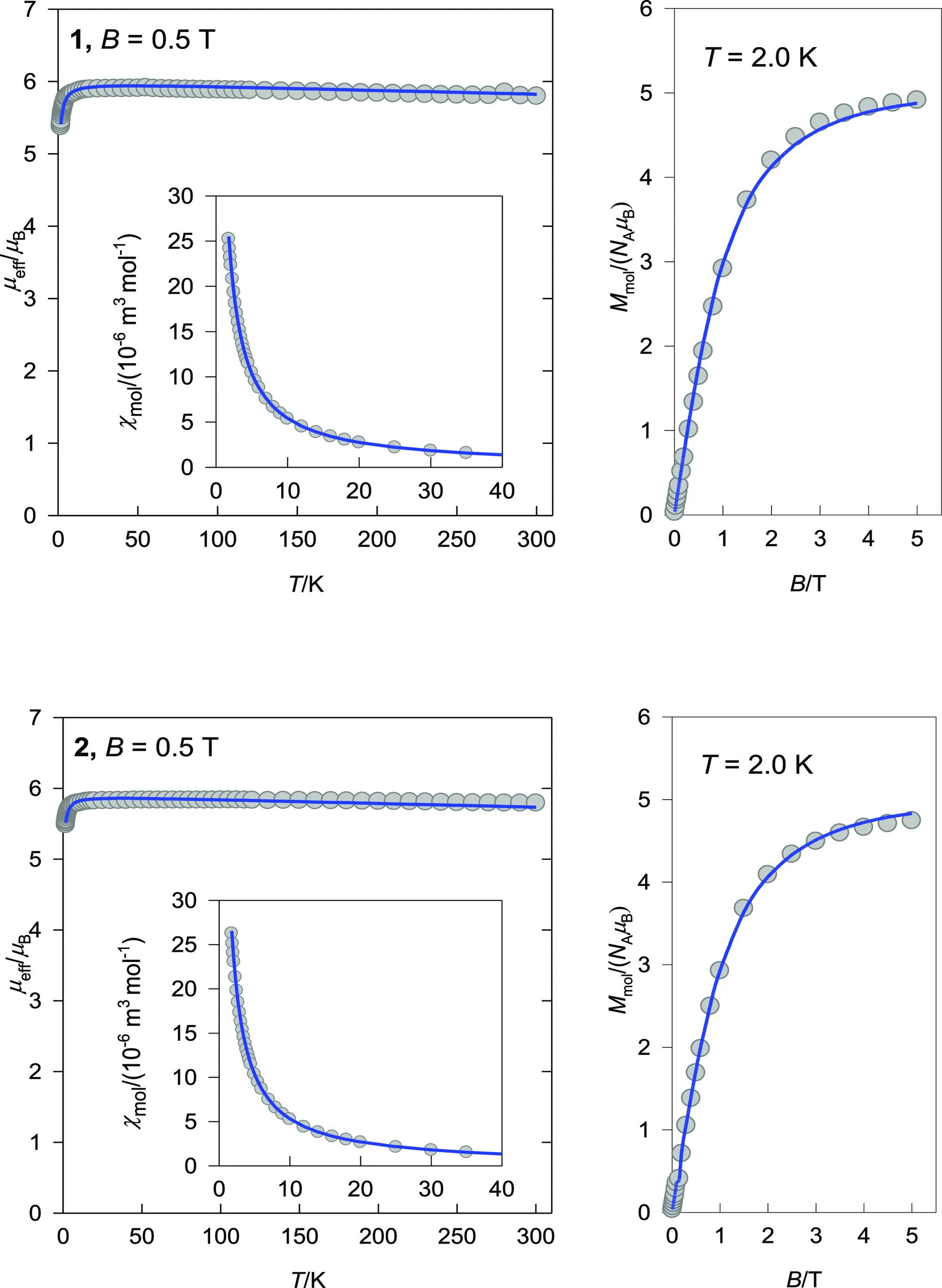
Magnetic functions for **(1)** and **(2)**. Solid lines—fitted on ZFS model.

Both magnetic data sets (susceptibility and magnetization) were fitted using a standard spin Hamiltonian that accounts for the axial zero-field splitting parameter *D* and the isotropic *g*-factor. Additionally, correction for the uncompensated temperature-independent magnetism (χ_TIM_) was employed. The fitting procedure utilized the minimization of the error functional *F* = *w*·*E*(χ)+(1-*w*)·*E*(*M*) where *E*(χ) and *E*(*M*) are relative errors of susceptibility and magnetization, respectively; it gave the magnetic parameters listed in Table 1. In accordance with expectation for ^6^A_1_ ground state, the calculated *g*-factors are 2.00 for **(1)** and 1.99 for **(2)**. Small *D*-values were retrieved; irrespective of their sign, such small values rule out the Orbach relaxation process as the primary mechanism for slow magnetic relaxation.

The DC magnetic data unambiguously confirm the presence of zero-field splitting. However, the quantitative determination of a small *D*-parameter can be problematic. DC magnetic data for Mn­(II) systems are sensitive to several influences. The true differential isothermal susceptibility is the derivative of magnetization with respect to magnetic field intensity. This is not exactly the magnetization divided by a small field, even when *B*_DC_ = 0.5 T. Moreover, the paramagnetic susceptibility is the measured value corrected for underlying diamagnetism and temperature-independent paramagnetism. The usual zero-field splitting model with *D* = 3*B*_2,0_ ignores the biquadratic contribution. Omission of *B*_2,2_ and *B*_4,0_ when modeling Mn­(II) magnetic susceptibility data can lead to inaccuracies because it neglects higher-order crystal-field distortions that affect the exact energy spacing. Note that the measured quantity is the magnetic moment of the specimen, which reflects the bulk properties of the substance, the presence of the container, and the instrument configuration. Therefore, the *D*-values derived for Mn­(II) systems from magnetometry should be considered with care. EPR is a completely different technique, so an exact match between EPR and magnetometric parameters is not always obtained.

### AC Magnetic Data

AC susceptibility measurements were recorded using a small alternating field *B*_AC_ = 0.03 mT with applied external fields *B*_DC_ = 0.01–0.8 T. Initial trials (Figure 4) at *T* = 2.0 K confirm that both complexes exhibit slow magnetic relaxation (SMR, or slow relaxation of magnetization) which is dependent on the alternating frequency *f*_AC_. The χ” values observed at *B*_DC_ = 0.30 (for **1**) and 0.35 T (for **2**) were selected for detailed mapping as shown below (Figure 4).

**Figure 4:**
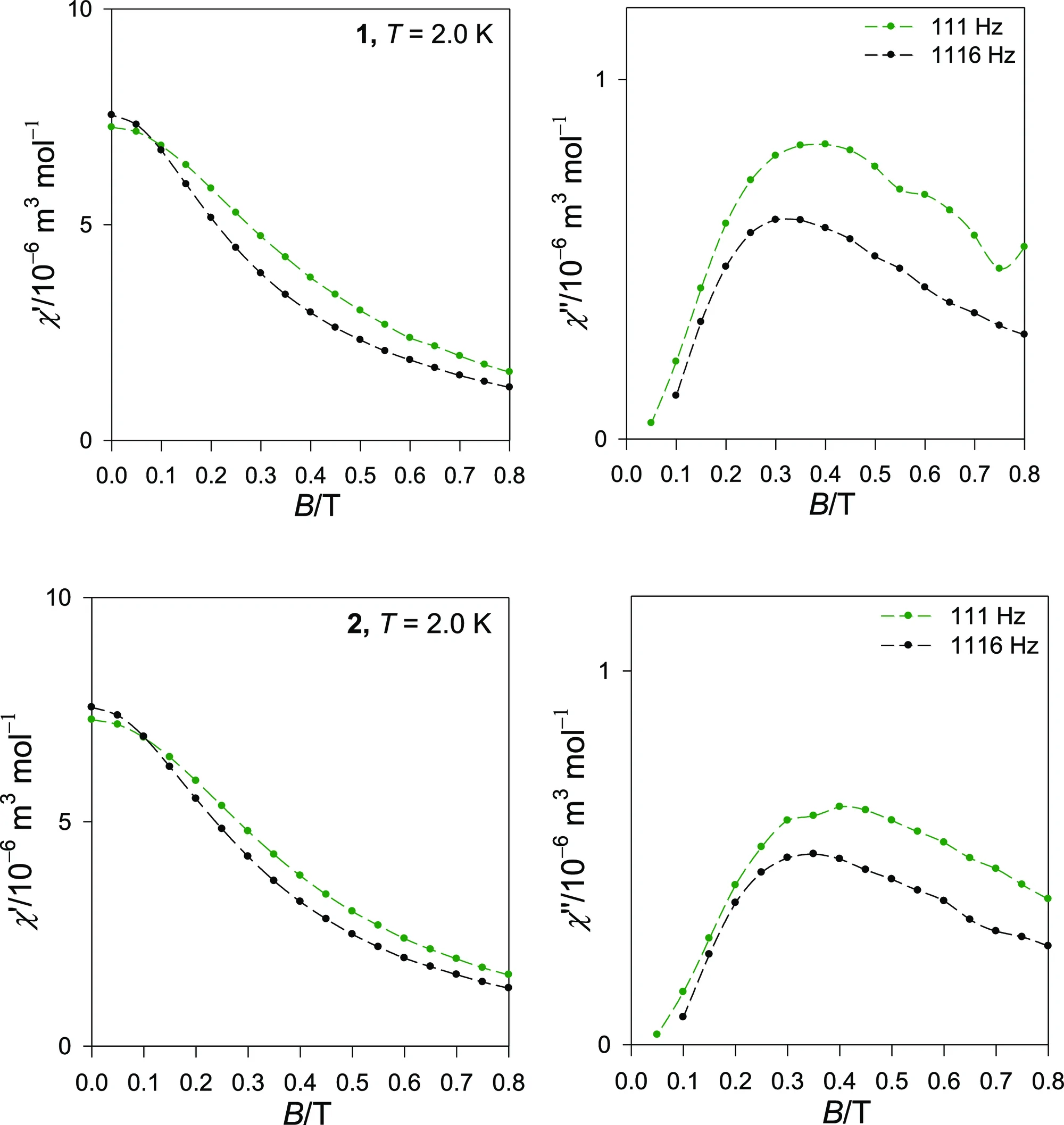
Field dependence of the AC susceptibility for two trial frequencies *f* = 111 and 1116 Hz at *T* = 2.0 K.

A temperature-dependent study of the AC susceptibility, with 22 frequencies ranging between *f* = 0.1–1500 Hz, shows that field supported SMR persists above *T* > 7 K. Prolonged data taking with two highest frequencies confirms that the out-of-phase susceptibility signal escapes at *T* ∼ 30 K (Figure S9).

The same data set was rearranged into graphs displayed in Figure 5 where the frequency dependence of the AC susceptibility is mapped for a set of temperatures between *T* = 2.0–7.0 K. Two maxima are seen at the ? vs *f* curve for **(1)** corresponding to two relaxation modes: the low-frequency (LF) and high-frequency (HF) mode. In contrast, complex **(2)** exhibits three relaxation modes, including an additional intermediate-frequency (IF) mode (close to HF). These data were fitted using the extended Debye model, which contains isothermal susceptibility χ_T*i*_, broadening factor α_*i*_, and relaxation time τ_*i*_ for each relaxation mode, plus a common adiabatic susceptibility increment χ_S_. The fitted data are provided in SI (Tables S9 and S10) along with standard deviations for individual parameters. These data allow reconstructing the envelope of the AC susceptibility which is drawn as a set of solid lines in Figure 5. Based on the fitted data, the Argand diagrams were constructed (Figure 6).

**Figure 5:**
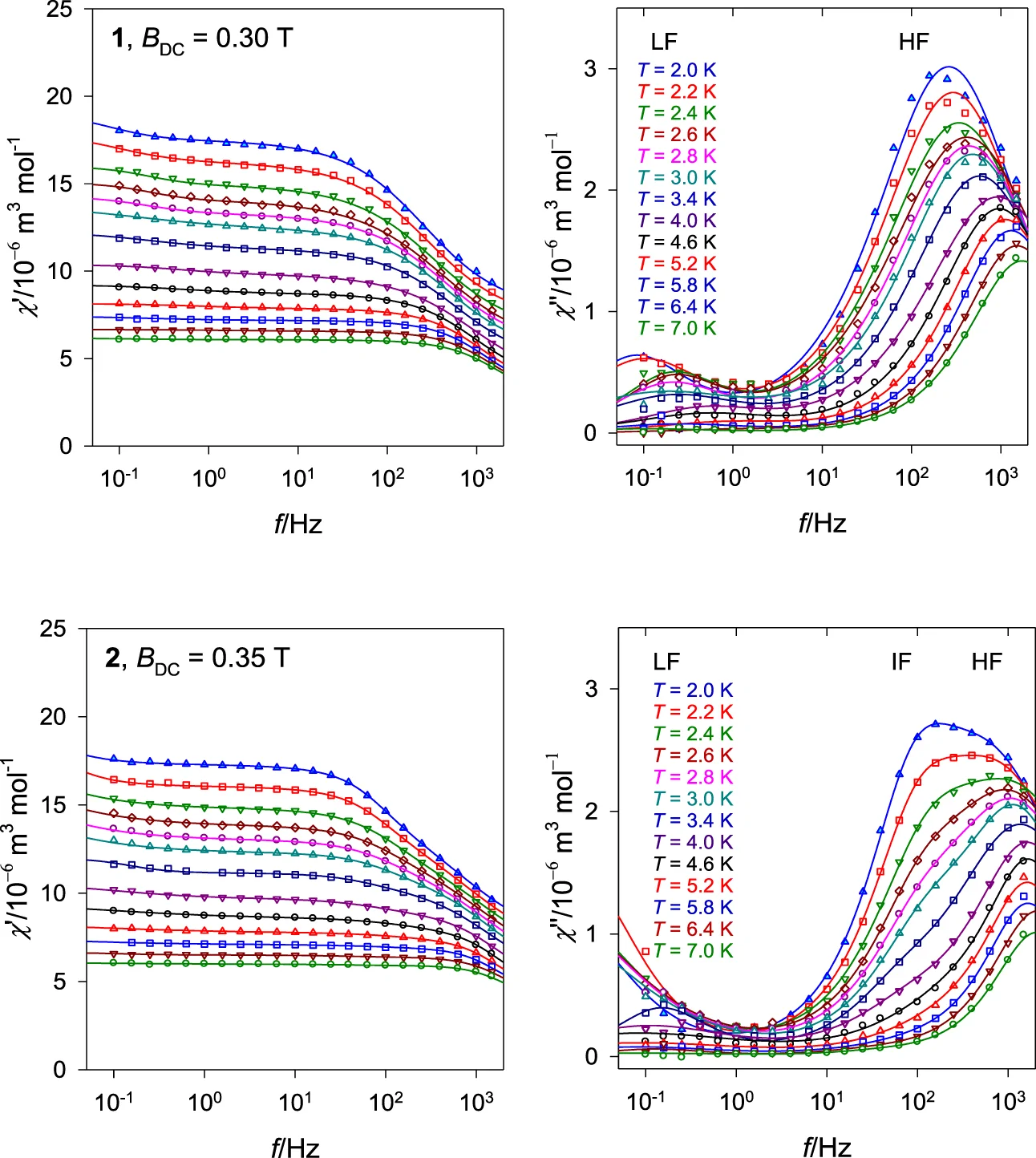
Frequency dependence of the AC susceptibility for varied temperature.

**Figure 6:**
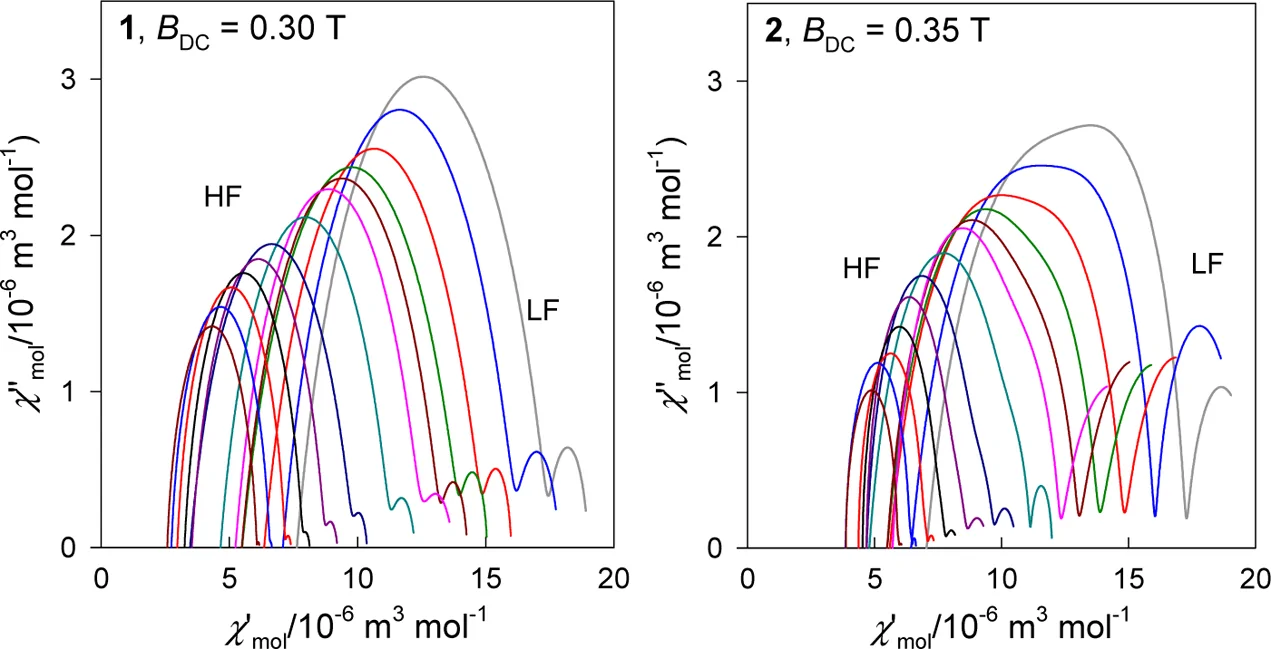
Argand plots for **(1)** and **(2)**.

The calculated relaxation times were arranged into a (classical) Arrhenius-like plot shown in Figure 7. No clear upturn toward slower relaxation was observed upon heating within the measurement range (up to 7 K).

**Figure 7:**
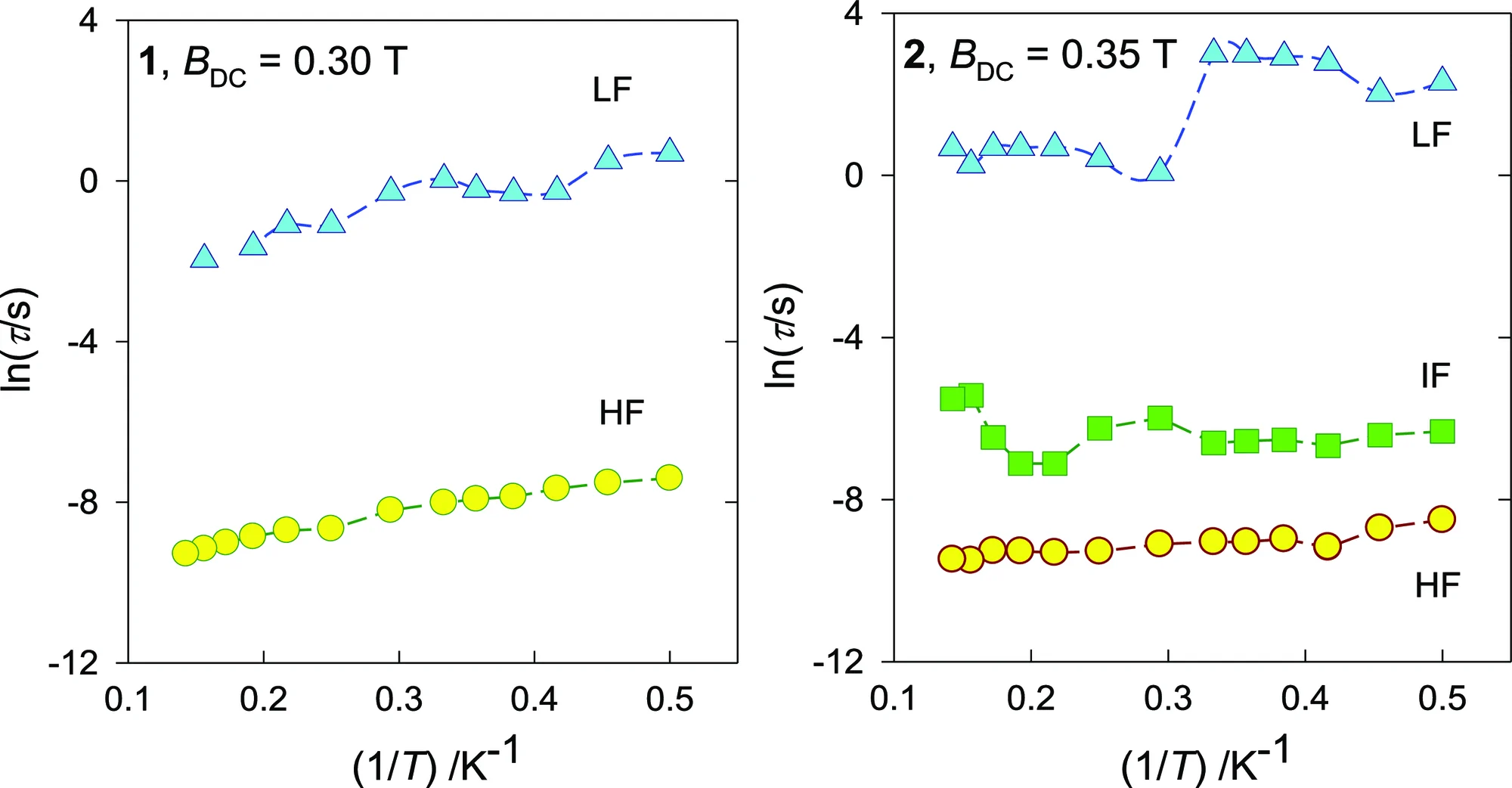
Arrhenius-like plot for **(1)** and **(2)**.

Further insight was gained from the lnτ­(HF) vs ln*T* plot (Figure 8). This allows a visualization of the linearized behavior τ^–1^ = *CT* ^*n*^, from which lnτ = – ln*C* – *n*·ln*T*; then the tangential defines the temperature exponent *n*. Based on the ln–ln plot in Figure 8, it is evident that the temperature coefficient is *n* = – *b*_1_ = 1.52 and 0.58. These values are inconsistent with Raman relaxation mechanisms, for which *n* = 5–9 is a typical range. Consequently, the slow magnetic relaxation in these complexes is attributed to direct processes (*n* ∼ 1) and/or photon bottleneck mechanisms (*n* ∼ 2) of slow magnetic relaxation.

**Figure 8:**
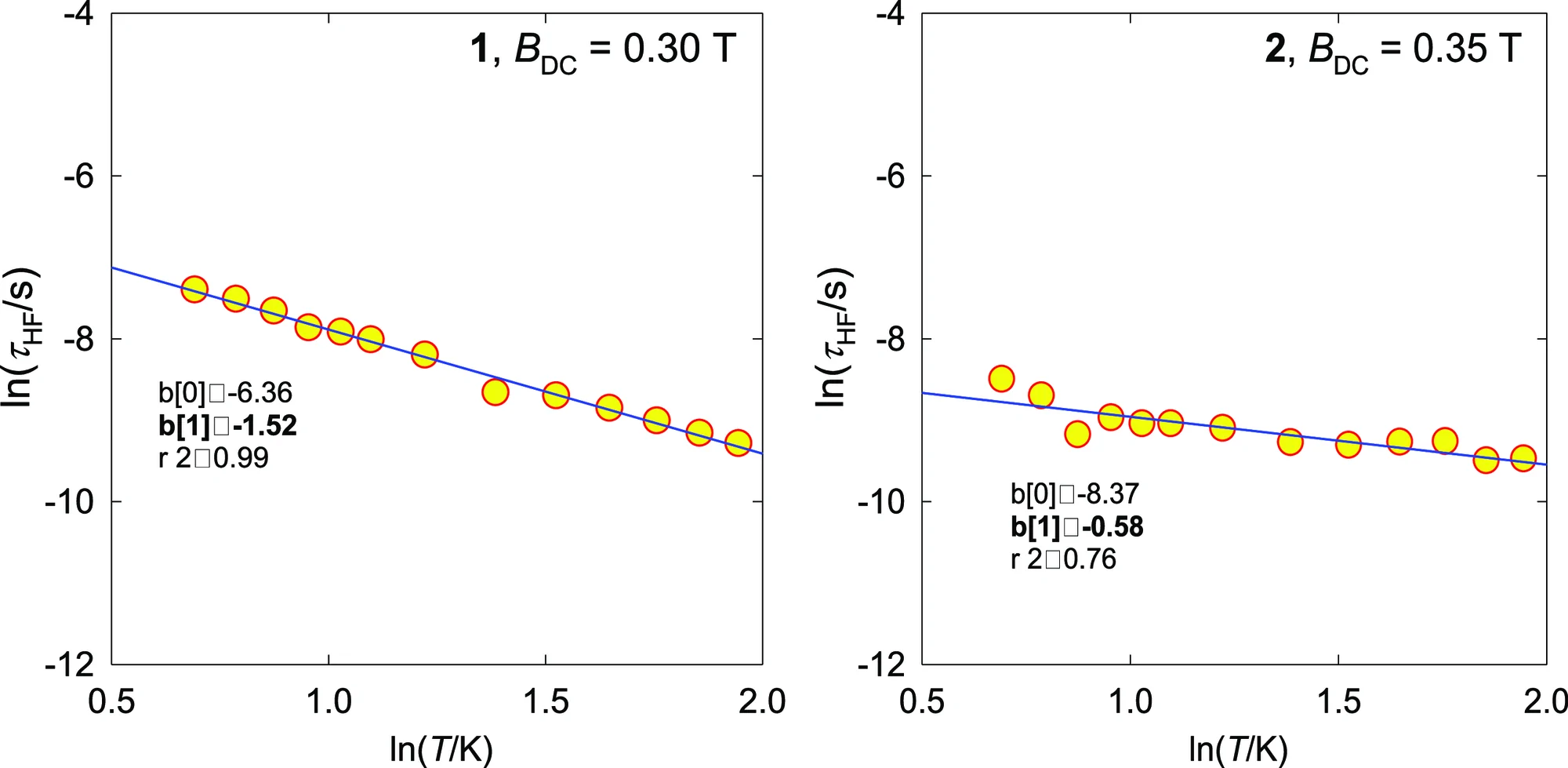
A ln–ln plot for the high-frequency relaxation time for **(1)** and **(2)**.

Slow magnetic relaxation in Mn­(II) complexes is a relatively rare phenomenon, with only a limited number of examples reported to date.^7–18^ The present study demonstrates that rare five-coordinate Mn­(II) complexes can exhibit slow magnetic relaxation despite the intrinsically low anisotropy of the *d* ^5^ electronic configuration.

The weak magnetic anisotropy, reflected by small *D* values derived from EPR and *ab initio* calculations, is therefore not sufficient to promote fast quantum tunnelling of magnetization (QTM) and suppress slow relaxation. In contrast to conventional octahedral Mn­(II) systems, the distorted square-pyramidal geometry leads to reduced symmetry and enhanced structural anisotropy. A key structural feature is the unusual tricoordinate mode with both nitrogen and oxygen donor atoms, which imposes a highly asymmetric ligand field. This rare coordination mode plays a decisive role in generating a finite zero-field splitting (*D*), placing the system in the quasi-isotropic regime. Importantly, the magnetic anisotropy does not originate from strong spin–orbit coupling but rather from geometric constraints associated with the low coordination number and ligand topology. As a consequence, even subtle structural distortions are sufficient to induce measurable anisotropy. The observed dynamic magnetic behavior is consistent with this picture, as slow relaxation proceeds via direct and phonon-mediated mechanisms rather than an Orbach process. The presence of multiple relaxation channels further reflects the structural and dynamical complexity of the system. Notably, to the best of our knowledge, no examples of slow magnetic relaxation have been previously reported for Mn­(II) complexes featuring this type of N_4_O coordination environment.

## Conclusions

In conclusion, two pentacoordinate Mn­(II) complexes containing pseudohalide coligands and an unusual {Mn^II^N_4_O} coordination environment were successfully synthesized and structurally characterized. Magnetic susceptibility, EPR spectroscopy, and ab initio calculations consistently revealed only very small magnetic anisotropy, confirming the quasi-isotropic nature of both systems. Nevertheless, AC susceptibility measurements demonstrated field-induced slow magnetic relaxation with two or three relaxation channels observable up to ca. 30 K, representing one of the broadest temperature ranges reported so far for Mn­(II)-based systems. The relaxation dynamics are governed predominantly by direct and phonon-mediated mechanisms rather than an Orbach process, indicating that large magnetic anisotropy is not a prerequisite for slow magnetic relaxation in Mn­(II) complexes. These results demonstrate that low coordination symmetry and unconventional ligand binding modes can provide an effective means of tuning magnetic anisotropy and relaxation in quasi-isotropic Mn­(II) systems, extending previous structure–property studies to the rare {Mn^II^ N_4_O} coordination environment and its associated multichannel relaxation dynamics.

## Supplementary Material



## Notes

Synthesis, additional structural, magnetic and EPR data, theoretical calculation (PDF)
