# Supplementary material for: Lowering Coordination Symmetry as a Strategy for Tuning Slow Magnetic Relaxation in Quasi-Isotropic Mn(II) Systems
Source: Inorg Chem. 2026 Sep 24;65(39):22986–95. doi: 10.1021/acs.inorgchem.6c02639 (PMC13644306; doi:10.1021/acs.inorgchem.6c02639)
Supplement: Supplementary file 1 [file ic6c02639_si_001.pdf]

Supporting Information

Lowering Coordination Symmetry as a Strategy for Tuning Slow Magnetic Relaxation in Quasi-Isotropic Mn(II) Systems

Jacek Sawka[a*], Barbara Machura[b], Roman Boča[c], Cyril Rajnák[c,d], , Maciej Witwicki[a], Dariusz Bieńko[e] Pavel Kopel[f], Alina Bieńko[a*]

[a] Faculty Of Chemistry, University of Wroclaw, Joliot-Curie 14, 50-383 Wrocław
[b] Institute of Chemistry, University of Silesia, 9th Szkolna St., 40-006 Katowice
[c] Faculty of Health Sciences, University of SS Cyril and Methodius 91701 Trnava 9, Czechia
[d] Faculty of Natural Sciences, University of SS Cyril and Methodius 91701 Trnava
[e] Faculty of Chemistry, Wroclaw University of Science Technology, Wybrzeze Wyspianskiego 27, Wroclaw 50-370, Poland.
[f] Department of Inorganic Chemistry, Faculty of Science, Palacky University in Olomouc, 17. listopadu 12, CZ-779 00 Olomouc, Czech Republic

Corresponding Authors

Jacek Sawka - email: jacek.sawka@uwr.edu.pl
Alina Bieńko - email: alina.bienko@uwr.edu.pl

Compounds under study

Mn(OMeBB)(NCS)$_2$ (1), $C_{18}H_{14}MnN_6OS_2$ (M = 449.41 g mol^{-1}), CCDC 2551333

Mn(OMeBB)(NCO)$_2$ (2), $C_{18}H_{14}MnN_6O_3$ (M = 417.29g mol^{-1}), CCDC 2551334

Supporting Information

1. Synthesis and characterization 3

Scheme S1. The ligand 2,2'-[oxybis(methylene)]bis(1H-1,3-benzimidazole) 3

2. IR studies 4

Figure S1. IR spectra of compound (**1**) 4

Figure S2. IR spectra of compound (**2**) 4

3. X-ray studies 4

Figure S3. The powder XRPD pattern of **(1)** (experimental - black) and the simulation of its powder pattern from the crystal structure (red). 4

Figure S4. The powder XRPD pattern of **(2)** (experimental - black) and the simulation of its powder pattern from the crystal structure (red). 5

4. Crystal structure determination and refinement 5

Table S1. Crystal data and structure refinement of **(1)** and **(2)** 5

Table S2. Selected bond lengths [Å] and angles [°] of **(1)** and **(2)** 6

Table S3. Distortion of the coordination sphere of metal ions in **(1)** and **(2)** from ideal five-vertexes polyhedral (pentagon, vacant octahedron, trigonal bipyramid, spherical square pyramid, and

Johnson trigonal bipyramid) calculated using the SHAPE program[2] along with the angular structural index parameter τ_5.[3]........ 7

Table S4. Five-coordinate pseudohalide manganese(II) coordination compounds deposited in the CSD (version 2025.2.0) along with Mn(II) complexes **(1)** and **(2)** discussed in the current work..... 8

Table S5. Short intra- and intermolecular hydrogen bonds detected in complexes **(1)** and **(2)**........10

Table S6. Short π•••π interactions with Cg(I)•••Cg(J) distances below 4 Å in the crystal structures **(1)** and **(2)**........11

Table S7. C—H•••Cg(J)(π-ring) interactions for complexes **(1)** and **(2)**........11

Figure S5. Crystal packing of **(1)** showing the formation of the hydrogen bonds between the hydrogens N–H of the benzimidazole rings and sulphur atoms of the thiocyanate ions. The detailed characteristics of these contacts are given in **Table S4**........12

Figure S6. Crystal packing of **(2)** showing the formation of the hydrogen bonds between the hydrogens N–H of the benzimidazole rings and oxygen atoms of the cyanate ions, along with C(10)–H(10A)•••O(2) short contacts. The detailed characteristics of these contacts are given in **Table S4**........12

Figure S7. Crystal packing of **(1)** showing π•••π interactions with Cg(I)•••Cg(J) distances below 4 Å. The detailed characteristics of these contacts are given in **Table S5**........13

Figure S8. Crystal packing of **(2)** showing π•••π interactions with Cg(I)•••Cg(J) distances below 4 Å along with X—H•••Cg(J)(π-ring) interactions. The detailed characteristics of these contacts are given in **Tables S5 and S6**........13

5. **Theoretical calculations**........13

Table S8. Selected theoretically calculated bond lengths [Å] and angles [°] of **(1)** and **(2).**

6. **Magnetic data**........14

Figure S9. Temperature evolution of the AC susceptibility for varied frequencies........15

Table S9. Temperature dependence of AC susceptibility parameters for **(1)** at B_{DC} = 0.30 T........15

Table S10. Temperature dependence of AC susceptibility parameters for **(2)** at B_{DC} = 0.35 T.[a]....16

7. **EPR spectra**........17

Figure S11. Dependence of the simulated X-band EPR spectra of complexes **1** (A) and **2** (B) on the rhombicity parameter *E/D*........17

Figure S11. Dependence of the simulated X-band EPR spectra of complexes **1** (A) and **2** (B) on the axial parameter |*D*|........18

8. **References**........19

1. Synthesis and characterization

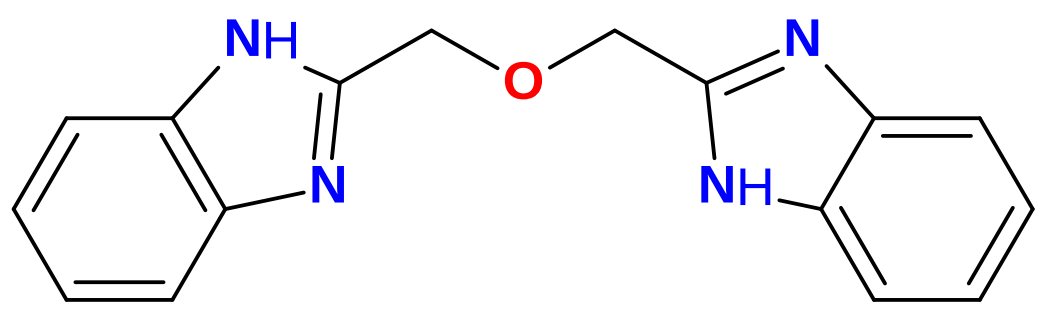

Scheme S1. The ligand 2,2'-[oxybis(methylene)]bis(1H-1,3-benzimidazole)

Preparation of the complexes

2,2'-[oxybis(methylene)]bis(1*H*-1,3-benzimidazole) (OMeBB) (**Scheme S1**) was synthesized according to the literature method.[1] The other reagents were commercially available and they were used without further purification. The air-stable manganese(II) complexes Mn(OMeBB)(NCS) **(1)** and Mn(OMeBB)(NCO) **(2)** were obtained as light brown crystals (for **(1)**) and white colors (for **(2)**).

Mn(OMeBB)(NCS)$_2$ (1)

KSCN (0.014 g, 0.21 mmol) dissolved in heat in methanol (1 cm^3) was slowly added to a beige methanol solution (1 cm^3) of OMeBB (0.01 g, 0.036 mmol) dissolved in heat. Dissolved salt $MnCl_2 \cdot 4H_2O$ (0.0072 g, 0.036 mmol) in methanol (1 cm^3) was added drop by drop into the resulting solution during continuous stirring for 5 min. The mother liquor was left to slow evaporation at room temperature and X-ray quality light brown prisms of **(1)** were formed after a few days. They were collected directly from the solution for measurements. Yield ca. 92 %.

IR (KBr/cm^{-1}): 2066(s) [ν(C=N$_{SCN}$)], 1431(vs), 1334(m), 1273(m) [ν(C=N)$_{OMeBB}$ and ν(C=C)$_{OMeBB}$];

Anal. Calc. for $C_{18}H_{14}MnN_6OS_2$ **(1)**: %C: 47.27; %H: 3.33; %N: 17.94; %S: 11.30;

Found: %C: 47.20; %H: 3.24; %N: 17.83; %S: 11.36

Mn(OMeBB)(NCO)$_2$ (2)

NaNCO (0.0093 g, 0.14 mmol) dissolved in water (0.5 cm^3) was slowly added to a white methanol solution (1 cm^3) of OMeBB (0.10 g, 0.036 mmol) dissolved in heat. Dissolved salt $MnCl_2 \cdot 4H_2O$ (0.072 g, 0.036 mmol) in methanol (1 cm^3) was added drop by drop into the resulting solution during continuous stirring for 5 min. The mother liquor was left to slow evaporation at room temperature and X-ray quality white prisms of **(2)** were formed after a few days. They were collected directly from the solution for measurements. Yield ca. 76 %.

IR (KBr/cm^{-1}): 2221(vs) [ν_{as}(C≡N)$_{NCO}$], 1386(s), 1232(s), 1103(m) [ν(C=N)$_{OMeBB}$ and ν(C=C)$_{OMeBB}$];

Anal. Calc. for $C_{18}H_{14}MnN_6O_3$ **(2)**: %C: 43.31; %H: 1.95; %N: 16.46;

Found: %C: 43.38; %H: 2.05; %N: 16.56

2. IR studies

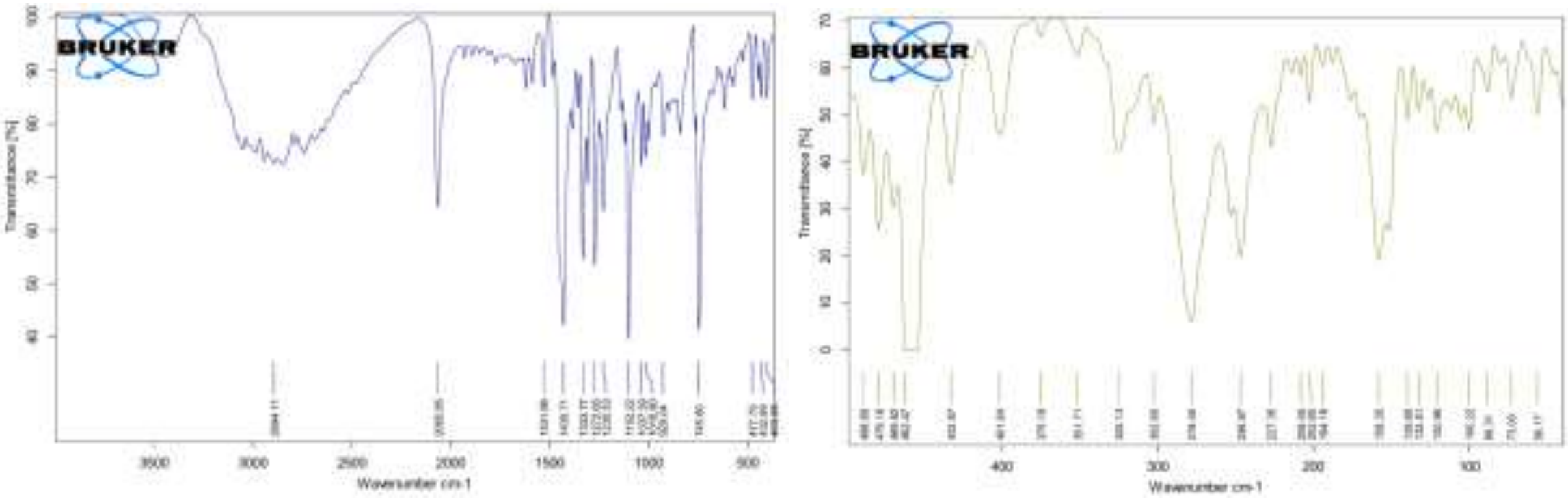

Figure S1. IR spectra of compound (**1**)

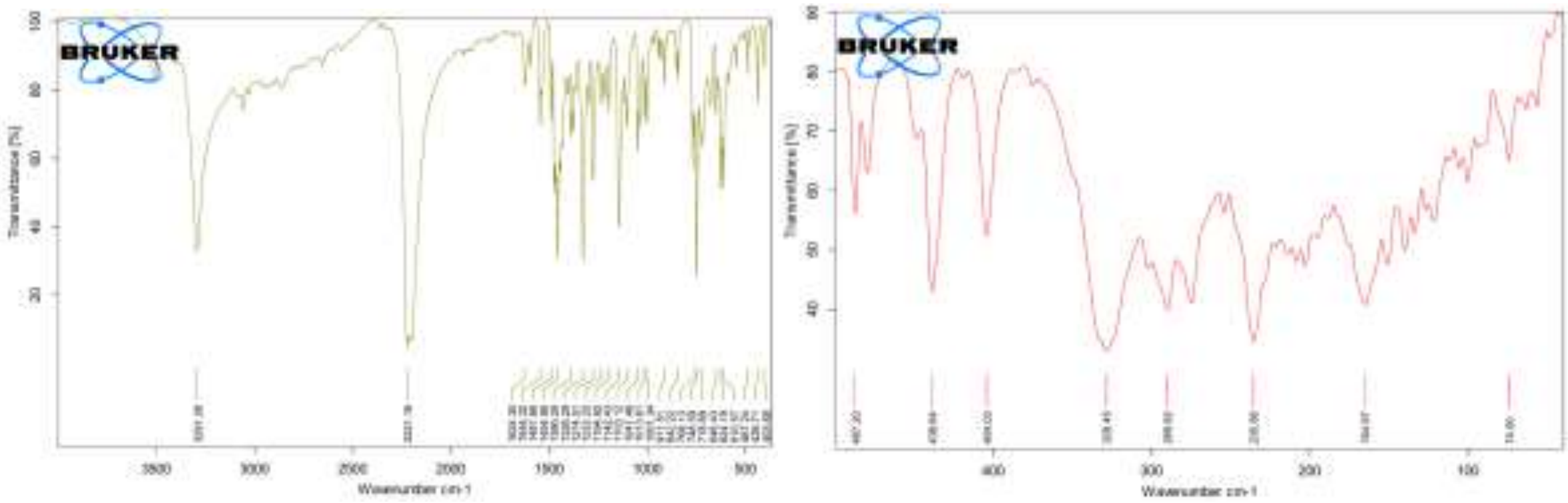

Figure S2. IR spectra of compound (**2**)

3. X-ray studies

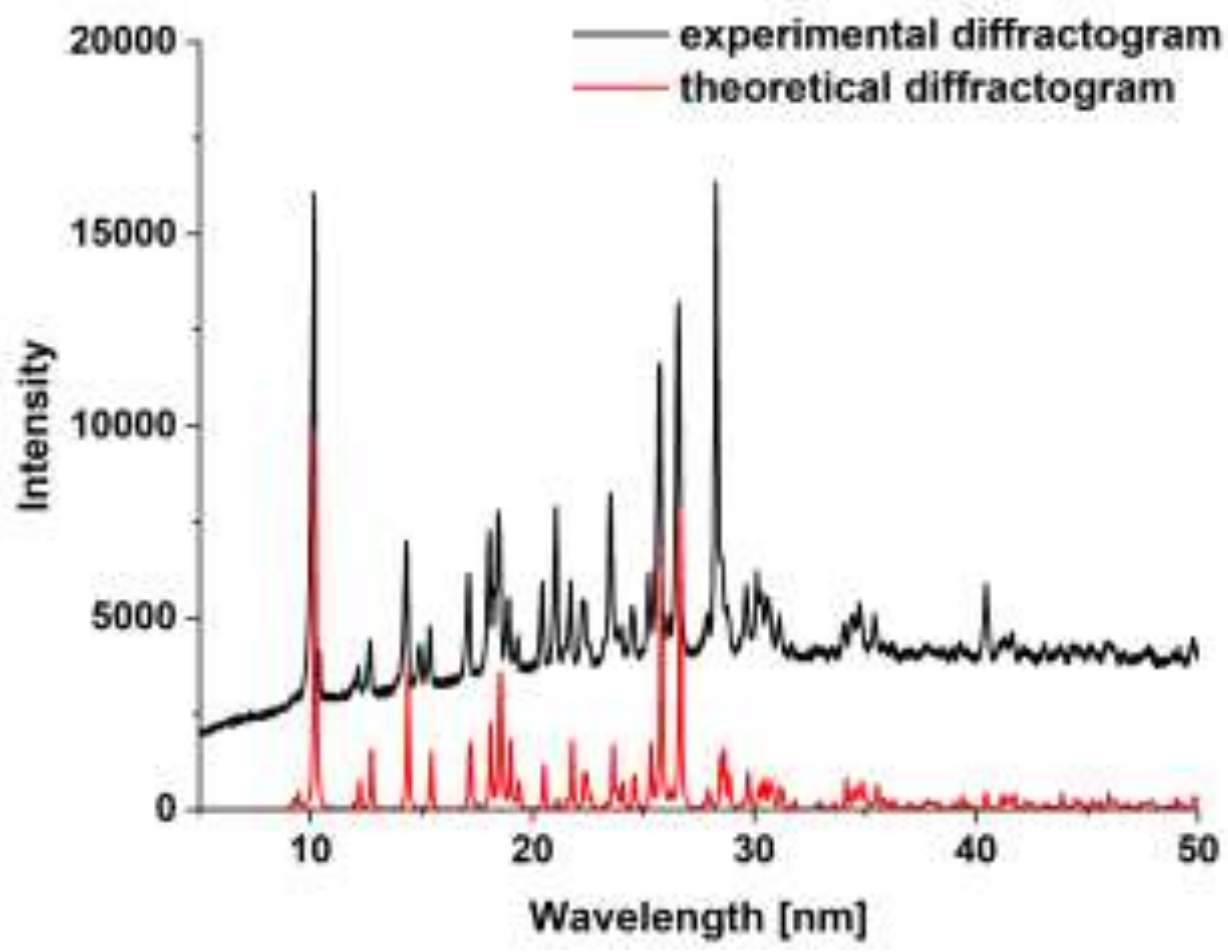

Figure S3. The powder XRPD pattern of **(1)** (experimental - black) and the simulation of its powder pattern from the crystal structure (red).

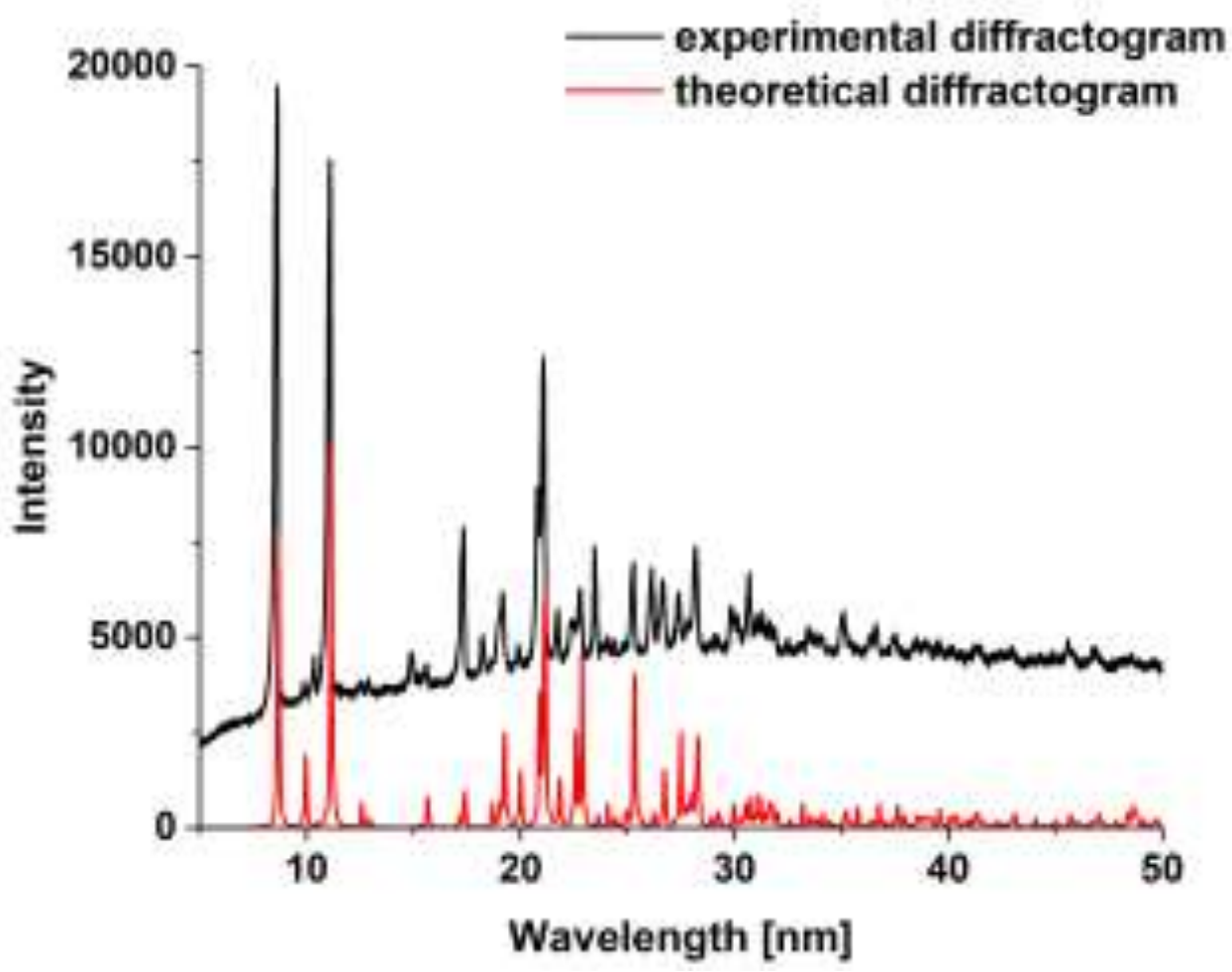

Figure S4. The powder XRPD pattern of **(2)** (experimental - black) and the simulation of its powder pattern from the crystal structure (red).

4. Crystal structure determination and refinement

Table S1. Crystal data and structure refinement of **(1)** and **(2)**.

Complex	(1)	(2)
**Empirical formula**	$C_{18}H_{14}MnN_6OS_2$	$C_{18}H_{14}MnN_6O_3$
**Formula weight**	449.41	417.29
**Temperature [K]**	293(2)	293(2)
**Wavelength [Å]**	0.71073	0.71073
**Crystal system**	Triclinic	Monoclinic
**Space group**	$P\bar{1}$	*C*2/*c*
**Unit cell dimensions [Å,°]**	a = 9.6204(6)	a = 16.0792(8)
	b = 10.5110(5)	b = 10.7070(6)
	c = 10.6739(6)	c = 20.6601(9)
	α = 65.666(5)	
	β = 76.715(5)	β = 99.649(5)
	γ = 80.707(4)	
**Volume [Å^3]**	954.42(10)	3506.5(3)
**Z**	2	8
**Density (calculated) [Mg/m^3]**	1.564	1.581
**Absorption coefficient [mm-1]**	0.932	0.787
**F(000)**	458.0	1704.0
**Crystal size [mm]**	0.3 × 0.19 × 0.035	0.345 × 0.165 × 0.10

**θ range for data collection [°]**	3.5 to 29.43	3.51 to 29.29
**Index ranges**	-12 ≤ h ≤ 11<br>-14 ≤ k ≤ 14<br>-14 ≤ l ≤ 14	-19 ≤ h ≤ 21<br>-14 ≤ k ≤ 12<br>-24 ≤ l ≤ 28
**Reflections collected**	9243	9503
**Independent reflections**	4485 (R_{int} = 0.0293)	4183 (R_{int} = 0.0256)
**Min. and max. transm.**	0.795 and 1.000	0.732 and 1.000
**Data / restraints / parameters**	4485/0/253	4183/0/253
**Goodness-of-fit on F^2**	1.037	1.062
**Final R indices [I>2σ(I)]**	R_1 = 0.0403<br>wR_2 = 0.0802	R_1 = 0.0361<br>wR_2 = 0.0864
**R indices (all data)**	R_1 = 0.0645<br>wR_2 = 0.0918	R_1 = 0.0477<br>wR_2 = 0.0927
**Largest diff. peak and hole [e Å^{-3}]**	0.30 and -0.28	0.24 and -0.33
**CCDC number**	2551333	2551334

Table S2. Selected bond lengths [Å] and angles [°] of **(1)** and **(2)**.

**(1)**		**(2)**	
**Bond lengths**			
Mn(1)–N(1)	2.085(2)	Mn(1)–N(1)	2.0658(18)
Mn(1)–N(2)	2.081(2)	Mn(1)–N(2)	2.0445(18)
Mn(1)–N(3)	2.1642(19)	Mn(1)–N(3)	2.1971(16)
Mn(1)–N(5)	2.1500(19)	Mn(1)–N(5)	2.1784(15)
Mn(1)–O(1)	2.3817(16)	Mn(1)–O(3)	2.3489(14)
N(1)–C(1)	1.155(3)	N(1)–C(1)	1.164(3)
N(2)–C(2)	1.159(3)	N(2)–C(2)	1.148(3)
C(1)–S(1)	1.624(3)	C(1)–O(1)	1.202(3)
C(2)–S(2)	1.622(3)	C(2)–O(2)	1.198(2)
**Bond angles**			
N(1)–Mn(1)–N(2)	109.09(9)	N(1)–Mn(1)–N(2)	110.83(8)
N(1)–Mn(1)–N(3)	95.46(8)	N(1)–Mn(1)–N(3)	107.90(7)
N(1)–Mn(1)–N(5)	110.26(9)	N(1)–Mn(1)–N(5)	105.81(7)
N(2)–Mn(1)–N(3)	102.30(8)	N(2)–Mn(1)–N(3)	99.08(7)
N(2)–Mn(1)–N(5)	101.19(8)	N(2)–Mn(1)–N(5)	99.46(7)

N(3)–Mn(1)–N(5)	136.80(7)	N(3)–Mn(1)–N(5)	132.23(6)
N(1)–Mn(1)–O(1)	113.82(8)	N(1)–Mn(1)–O(3)	107.90(6)
N(2)–Mn(1)–O(1)	136.70(8)	N(2)–Mn(1)–O(3)	141.26(7)
N(3)–Mn(1)–O(1)	68.64(6)	N(3)–Mn(1)–O(3)	69.00(5)
N(5)–Mn(1)–O(1)	69.35(6)	N(5)–Mn(1)–O(3)	69.03(5)
Mn(1)–N(1)–C(1)	154.5(2)	Mn(1)–N(1)–C(1)	166.73(18)
Mn(1)–N(2)–C(2)	162.3(2)	Mn(1)–N(2)–C(2)	165.63(18)
N(1)–C(1)–S(1)	179.1(2)	N(1)–C(1)–O(1)	179.2(2)
N(2)–C(2)–S(2)	178.4(2)	N(2)–C(2)–O(2)	177.2(2)

Table S3. Distortion of the coordination sphere of metal ions in **(1)** and **(2)** from ideal five-vertexes polyhedral (pentagon, vacant octahedron, trigonal bipyramid, spherical square pyramid, and Johnson trigonal bipyramid) calculated using the SHAPE program[2] along with the angular structural index parameter τ_5.[3]

**Compound**	**SHAPE v2.0 Continuous Shape Measures**					**τ_5**
	**Pentagon PP-5 (D5h)**	**Vacant octahedron vOC-5 (C4v)**	**Trigonal bipyramid TBPY-5 (D3h)**	**Spherical square pyramid SPY-5 (C4v)**	**Johnson trigonal bipyramid J12 JTBPY-5 (D3h)**	
**1**	26.245	5.270	5.119	**2.922**	9.094	0.002
**2**	29.383	4.326	6.489	**1.730**	10.037	0.150

Continuous Shape Measures are calculated with use of SHAPE v2.0 employing eq. 1:

$$S_Q(P) = min\left[\frac{\sum_{i=1}^{n}|\vec{q}_i-\vec{p}_i|^2}{\sum_{i=1}^{n}|\vec{q}_i-\vec{q}_0|^2}\right] \times 100 \qquad (1)$$

where $\vec{q_i}$ – N vectors with the 3N Cartesian coordinates of the problem structure Q, $\vec{p_i}$ – the coordinates of the ideal polyhedron P, $\vec{q_0}$ – the position vector of the geometric center that is chosen to be the same for the two polyhedral.

Parameter τ_5 is calculated with use of the eq. 2:

$$\tau_5 = \frac{\beta - \alpha}{60°} \qquad (2)$$

where α and β are two greatest valence angles, $\alpha < \beta$.

Table S4. Five-coordinate pseudohalide manganese(II) coordination compounds and complex ions deposited in the CSD (version 2025.2.0) along with Mn(II) complexes (**1**) and (**2**) discussed in the current work.

Refcode	Compound	Addison parametr τ	Ref.
**1**	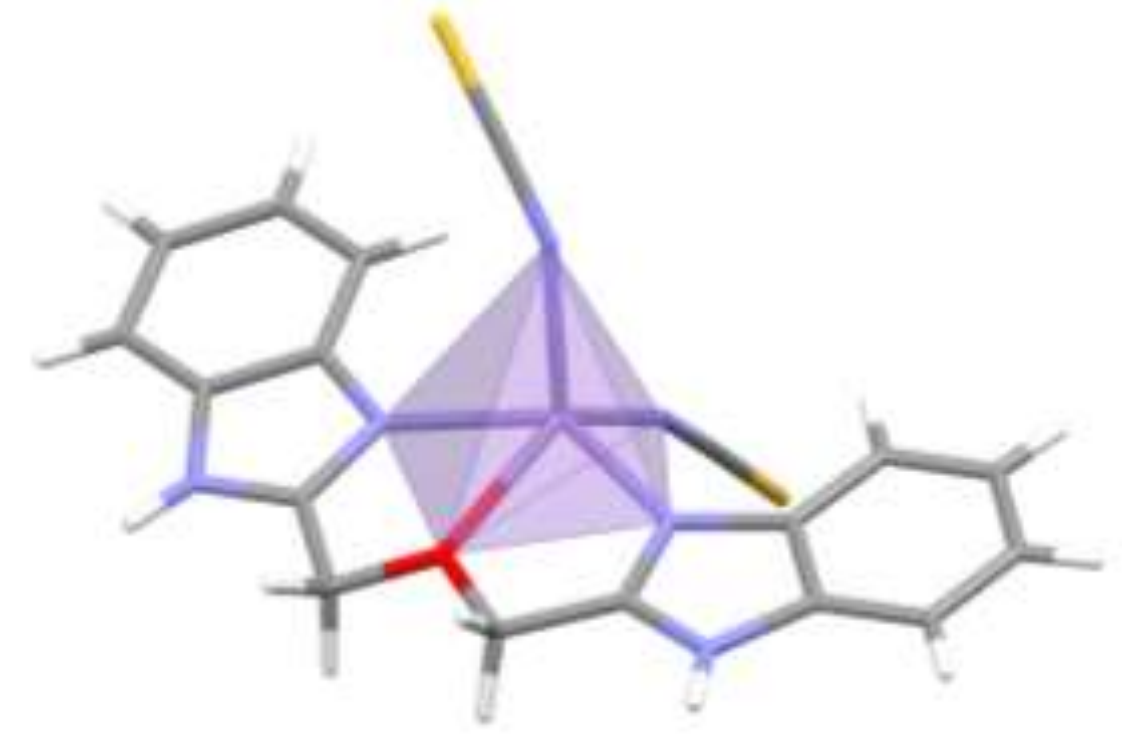	0.002	This work
**2**	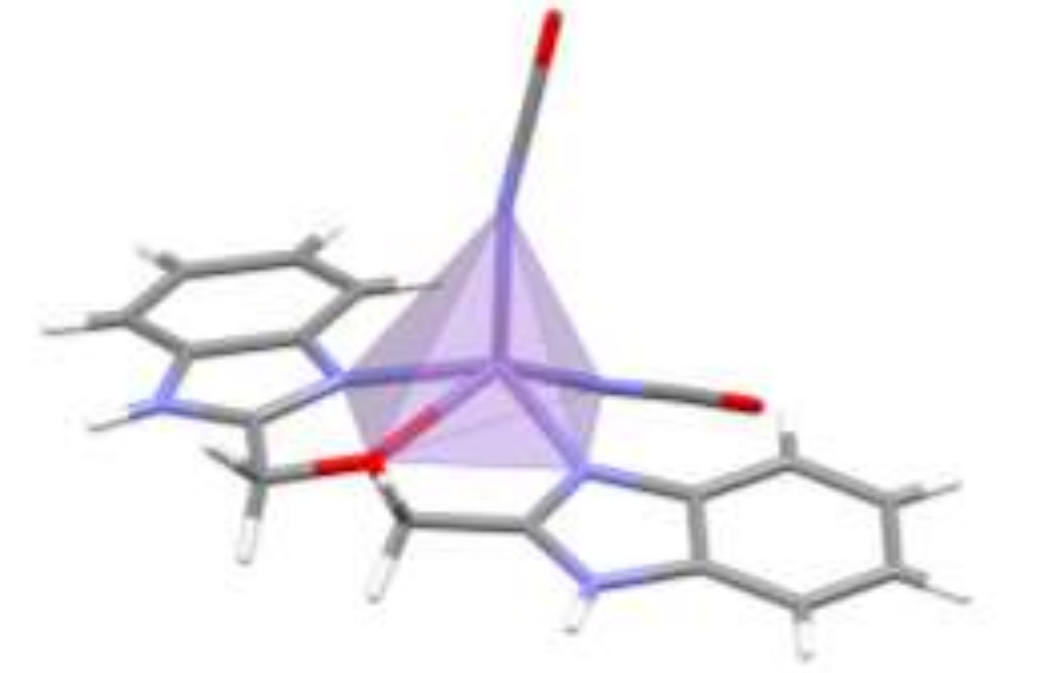	0.150	This work
**MIVYID**	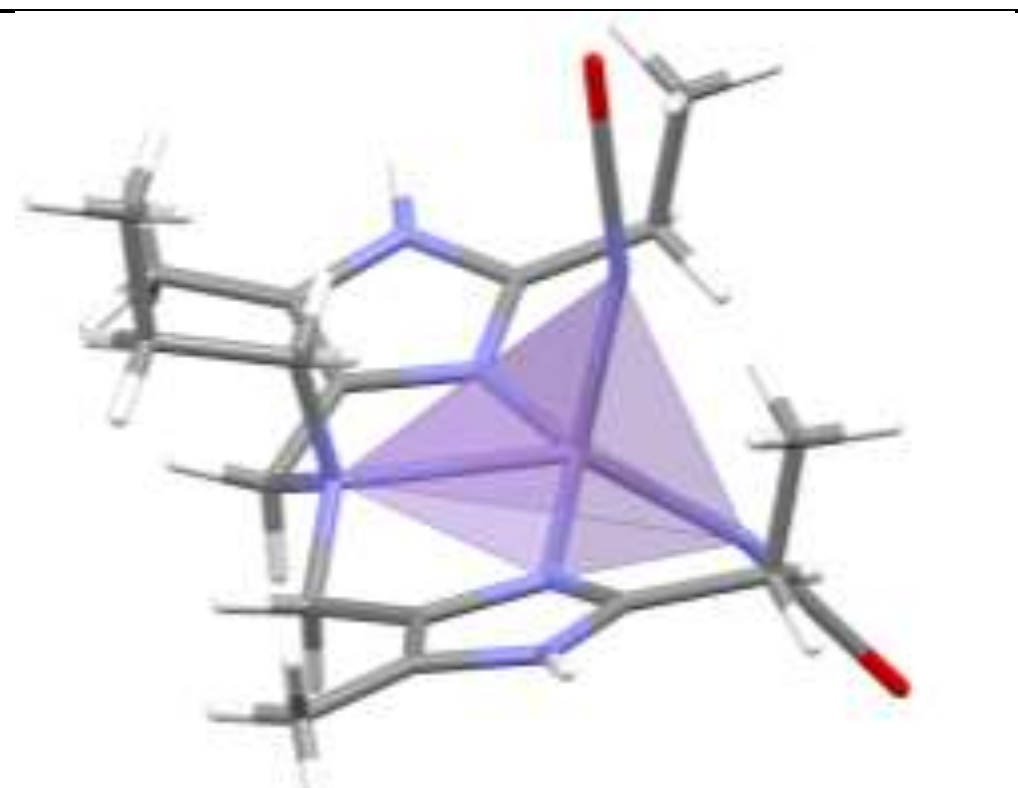	0.035	4
**BOFNAN**	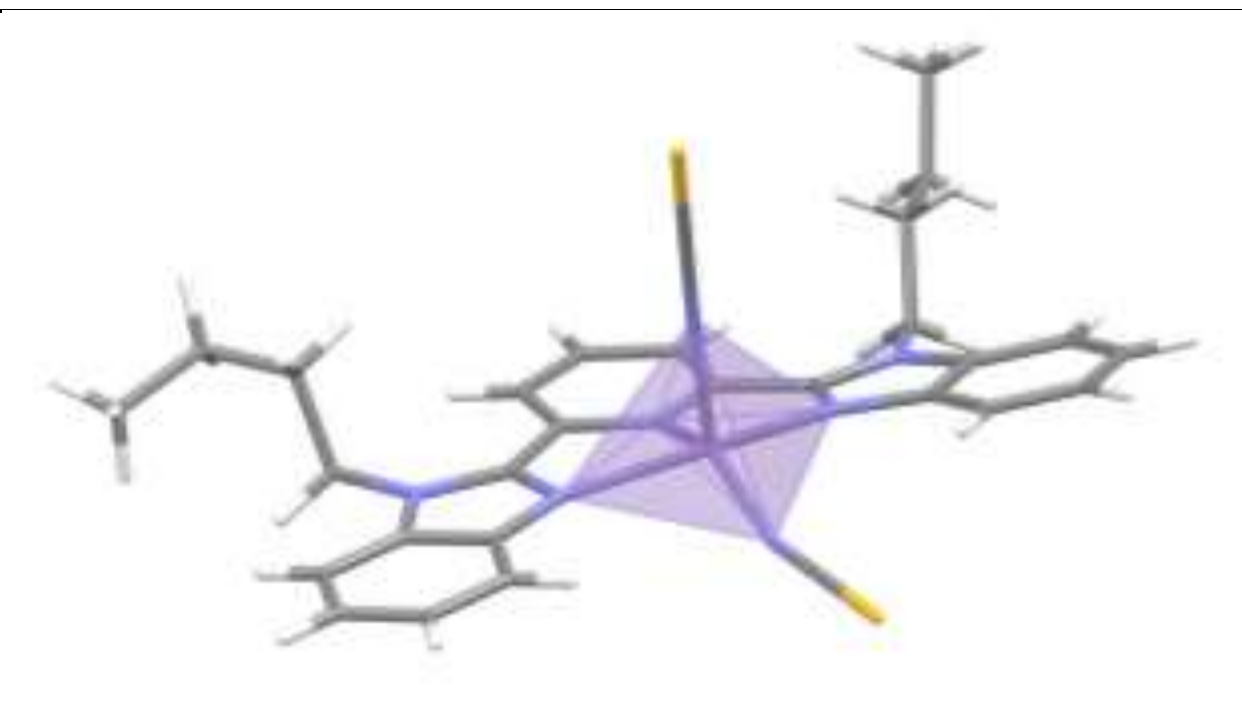	0.092	5

**OKUXUO**	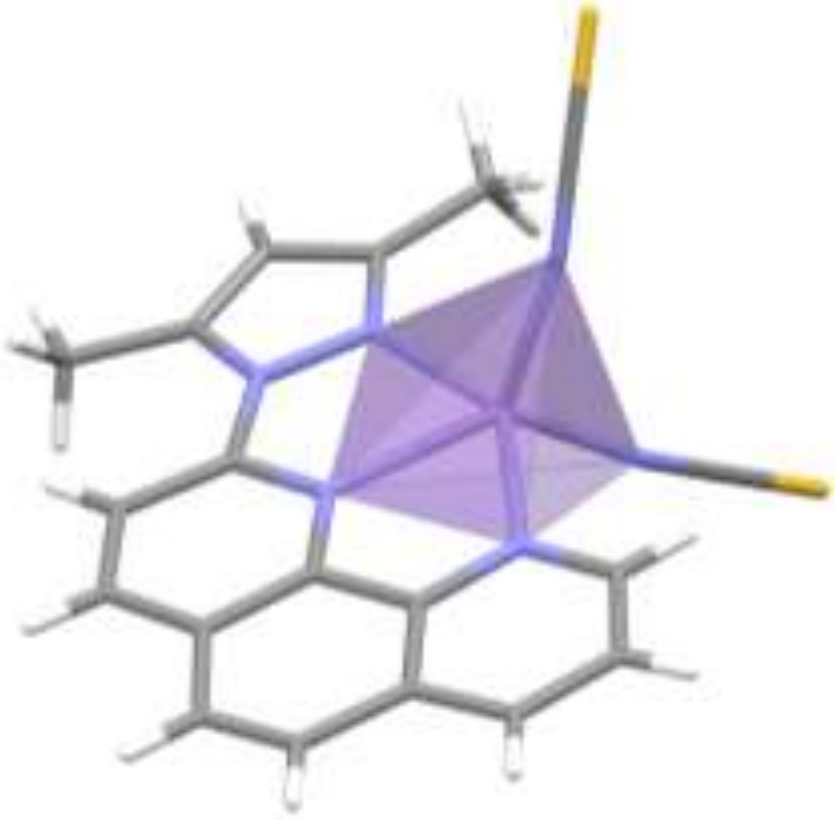	**O.218**	6
**HAHYEV**	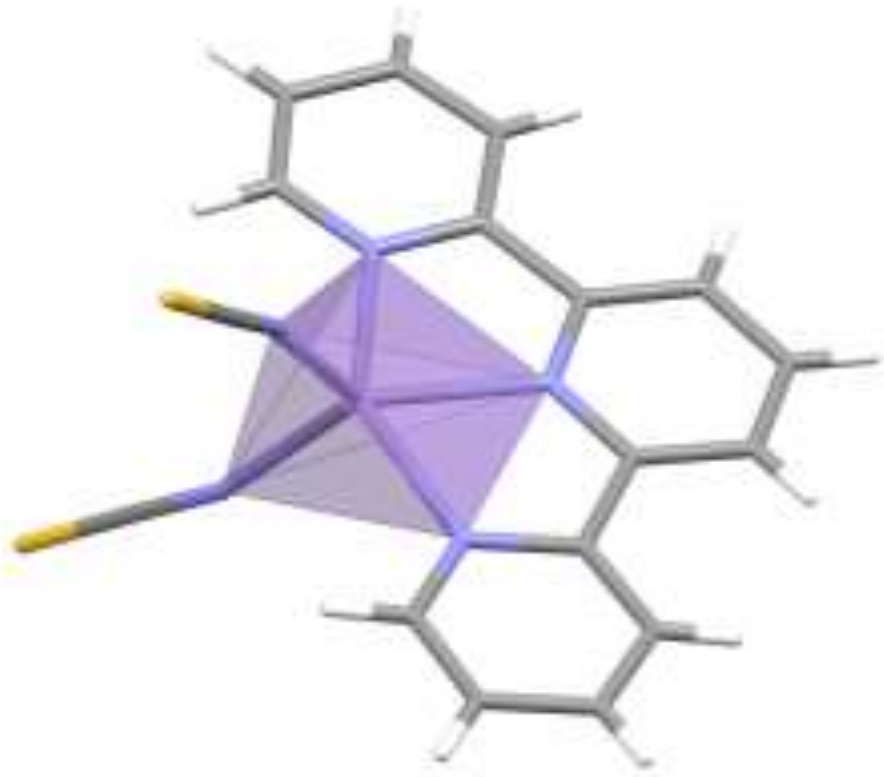	**0.290**	7
**ORIVAO**	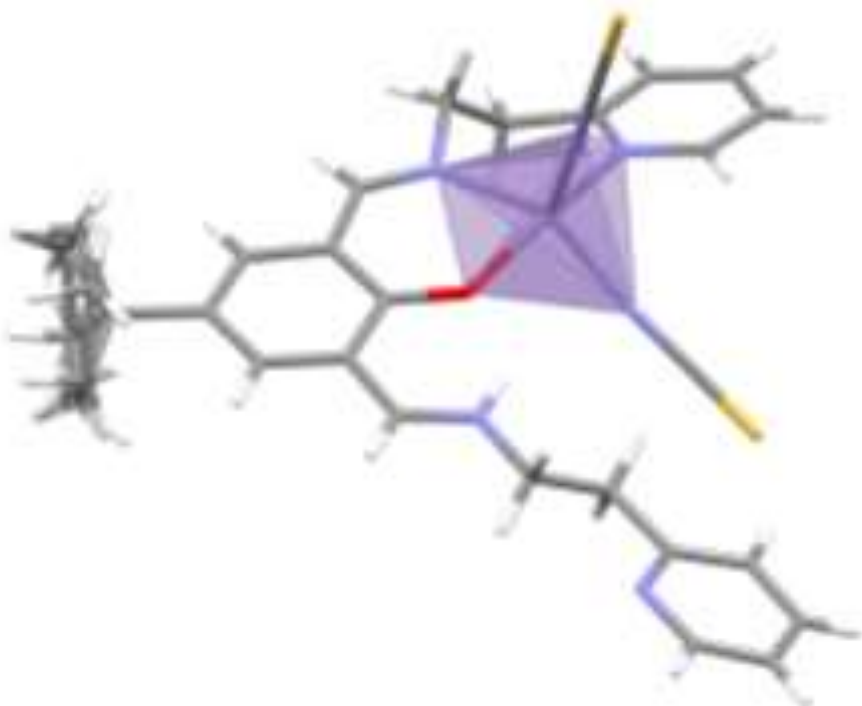	**0.332**	8
**ZEYWAD**	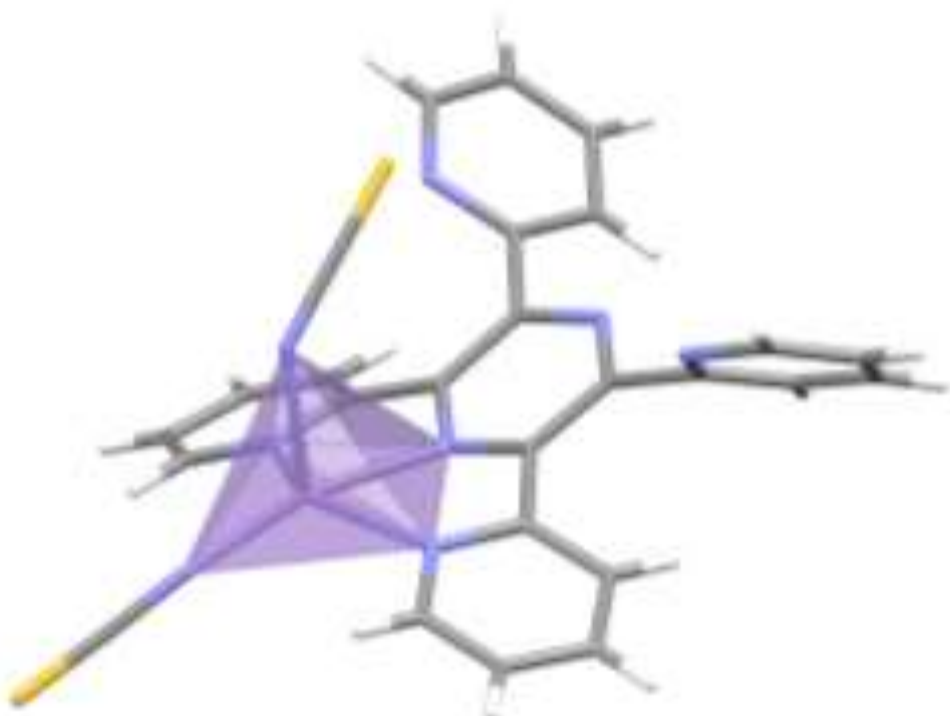	**0.363**	9

**FILKIW**	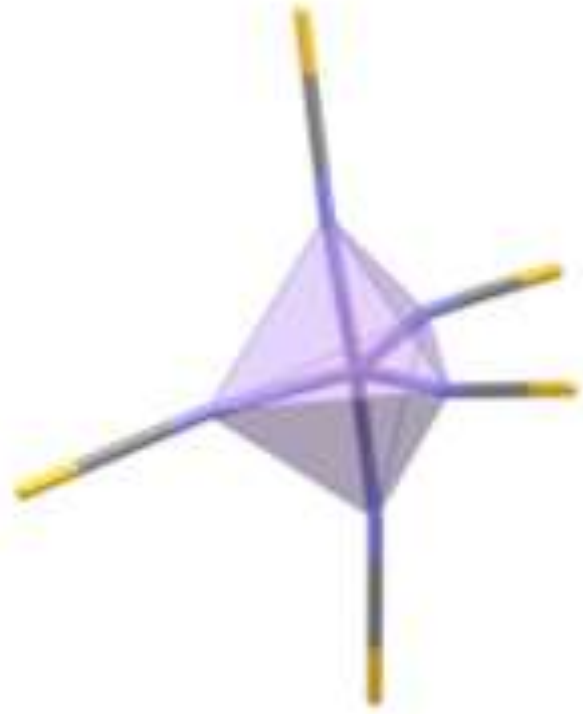	**0.618**	10
**TERVOE**	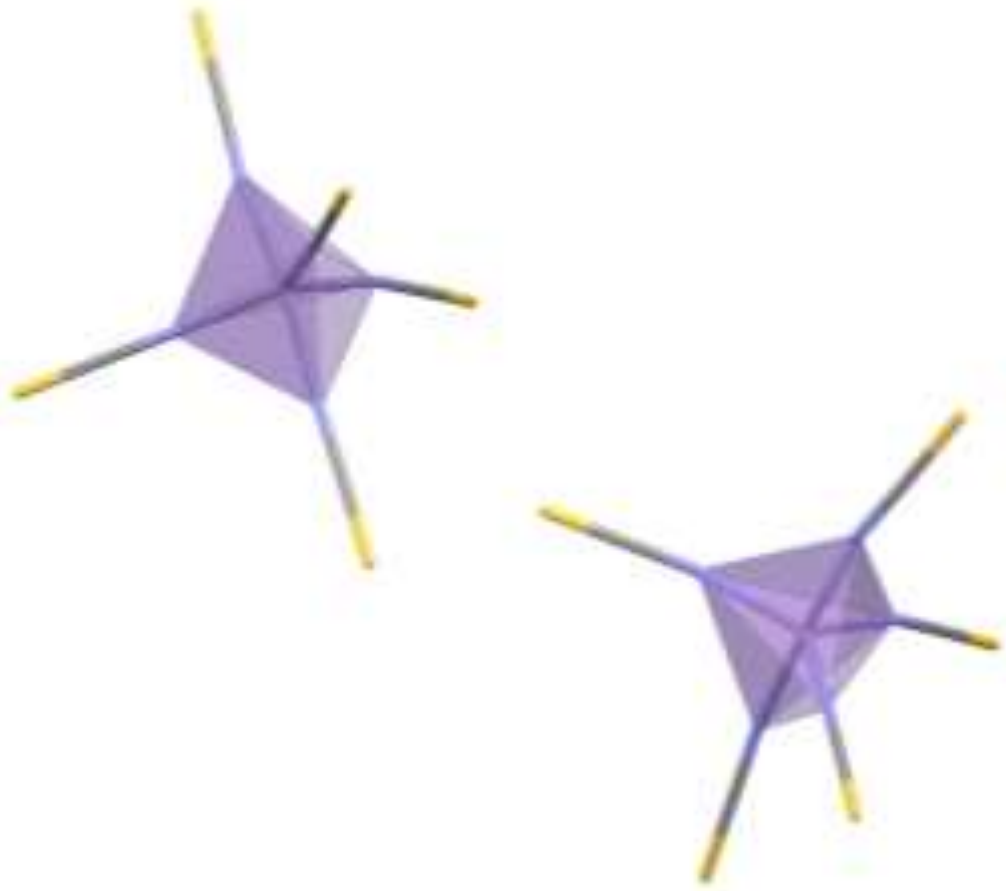	**0.851**<br>**0.918**	11
**XUHCUA**	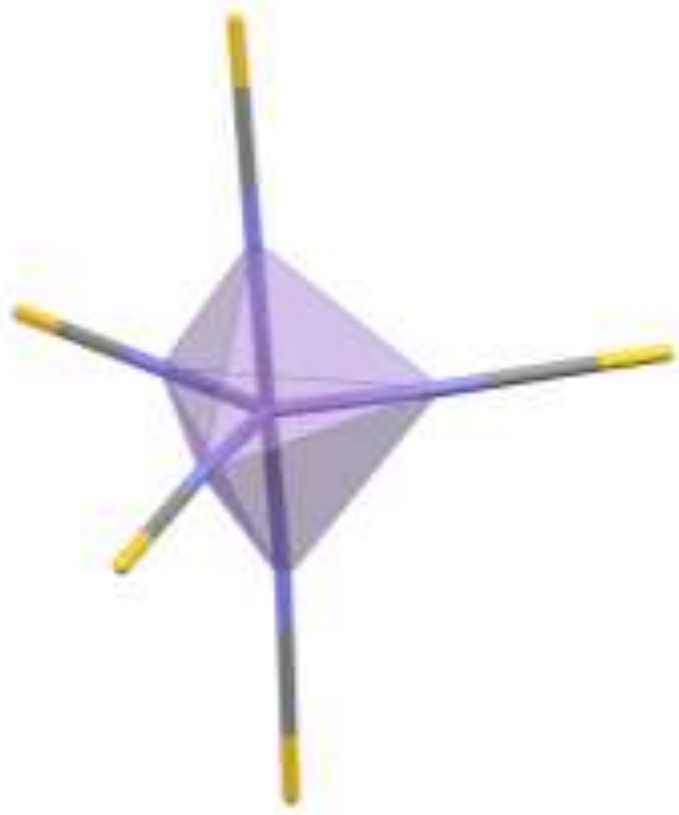	**0.981**	8

Table S5. Short intra- and intermolecular hydrogen bonds detected in complexes **(1)** and **(2)**.

D–H•••A	D–H [Å]	H•••A [Å]	D–A [Å]	D–H•••A [°]
**(1)**				
N(4)–H(4)•••S(1)[a]	0.86	2.51	3.328(2)	159.00
N(6)–H(6)•••S(2)[b]	0.86	2.67	3.445(2)	141.00
**(2)**				
N(4)–H(4)•••O(1)[c]	0.86	2.30	3.064(3)	148.00

N(4)–H(4)•••O(1)[d]	0.86	2.51	3.110(2)	128.00
N(6)–H(6)•••O(2)[e]	0.86	2.11	2.796(2)	136.00
C(10)–H(10A)•••O(2)[f]	0.97	2.46	3.415(3)	167.00

symmetry codes: (a) = -x,2-y,1-z; (b) = 1-x,1-y,2-z; (c) = 1/2+x,1/2+y,z; (d) = 3/2-x,1/2+y,3/2-z; (e) = 1/2+x,-1/2+y,z; (f) = 3/2-x,3/2-y,1-z

Table S6. Short π•••π interactions with Cg(I)•••Cg(J) distances below 4 Å in the crystal structures **(1)** and **(2)**.

Cg(I)•••Cg(J)	Cg(I)•••Cg(J) [Å]	α [°]	β [°]	γ [°]	Cg(I)-Perp [Å]	Cg(J)-Perp [Å]
**(1)**						
Cg(3)•••Cg(3)[a]	3.7427(13)	0	20.64	20.64	-3.5026(9)	-3.5026(9)
Cg(3)•••Cg(5)[a]	3.9814(15)	0.31(13)	28.33	28.37	-3.5033(9)	-3.5047(11)
Cg(4)•••Cg(4)[a]	3.7109(13)	0	15.10	15.10	3.5827(9)	3.5827(9)
Cg(4)•••Cg(5)[b]	3.9269(14)	7.96(13)	25.50	32.48	3.3125(9)	-3.5445(11)
Cg(4)•••Cg(6)[c]	3.8438(14)	1.15(12)	20.41	21.56	3.5749(9)	3.6024(11)
Cg(5)•••Cg(6)[d]	3.5518(16)	7.36(13)	2.95	6.17	-3.5313(11)	3.5472(11)
**(2)**						
Cg(4)•••Cg(4)[e]	3.4723(11)	0	23.19	23.19	-3.2785(8)	-3.2785(8)
Cg(4)•••Cg(5)[e]	3.5939(12)	15.86(10)	10.97	8.37	-3.5555(7)	3.5281(9)

symmetry codes: (a) = -x,2-y,1-z; (b) = 1+x,y,z; (c) = 1-x,1-y,2-z; (d) = -1+x,y,z; € = 3/2-X,1/2-Y,1-Z;
α = dihedral angle between *Cg*(I) and *Cg*(J); *Cg*(I)-Perp = Perpendicular distance of *Cg*(I) on ring J; *Cg*(J)-Perp = perpendicular distance of *Cg*(J) on ring I; β = angle *Cg*(I)→*Cg*(J) vector and normal to ring I; γ = angle *Cg*(I) →*Cg*(J) vector and normal to plane J. Cg(3) is the centroid of atoms = N3/C3/C8/N4/C9; Cg(4) is the centroid of atoms = N5/C12/N6/C13/C18; Cg(5) is the centroid of atoms = C3/C4/C5/C6/C7/C8; Cg(6) is the centroid of atoms = C13/C14/C15/C16/C17/C18

Table S7. C—H•••Cg(J)(π-ring) interactions for complexes **(1)** and **(2)**.

C—H•••Cg(J)	H•••Cg(J) [Å]	X•••Cg(J) [Å]	H–Perp [Å]	γ [°]	X(I)-H•••Cg(J) [°]
**(1)**					
no interactions detected					
**(2)**					
C(6)—H(6A)•••Cg(3)[a]	2.93	3.781(2)	2.74	20.55	153
C(11)—H(11A)•••Cg(6)[a]	2.70	3.505(2)	-2.67	7.97	141

symmetry codes: (a) = 3/2-x,1/2+y,3/2-z; (b) = 3/2-x,1/2-y,1-z; γ = angle X(I)→Cg(J) vector and normal to plane J. Cg(3) is the centroid of atoms = N3/C3/C8/N4/C9;; Cg(6) is the centroid of atoms = C13/C14/C15/C16/C17/C18;

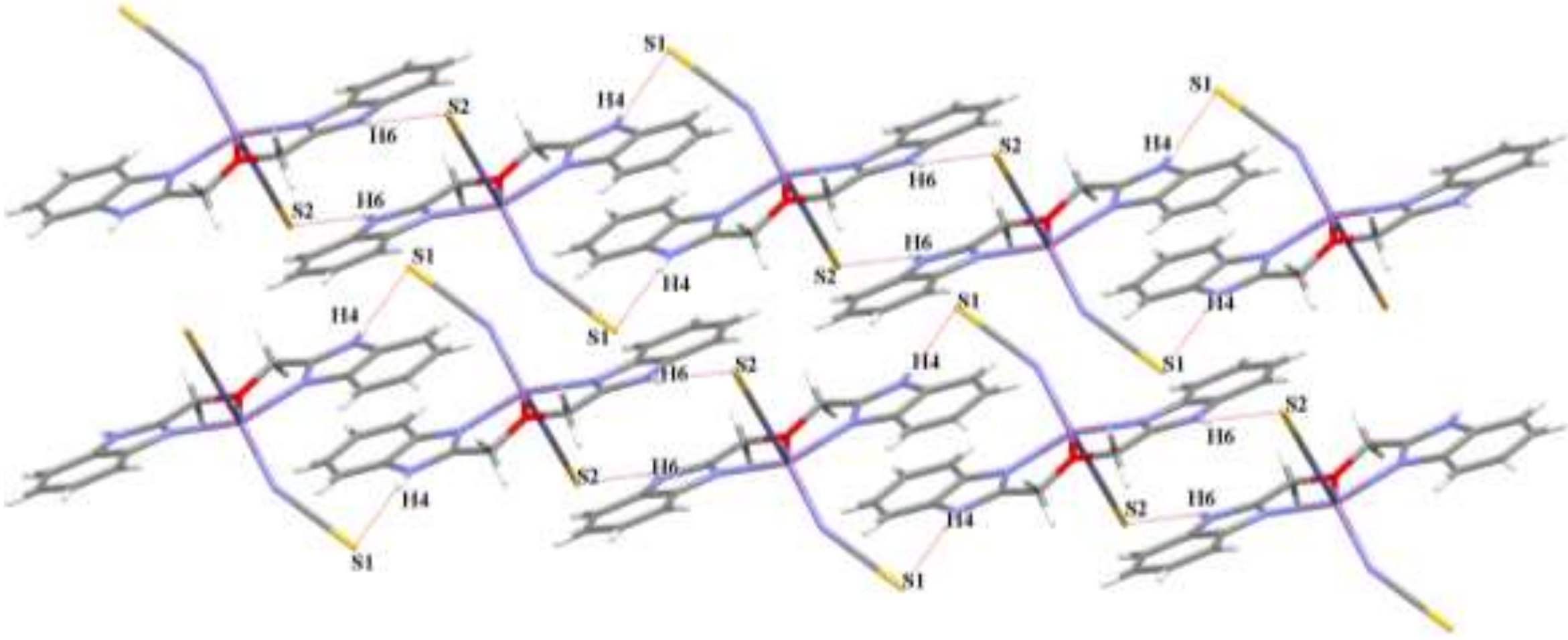

Figure S5. Crystal packing of **(1)** showing the formation of the hydrogen bonds between the hydrogens N–H of the benzimidazole rings and sulphur atoms of the thiocyanate ions. The detailed characteristics of these contacts are given in **Table S4**.

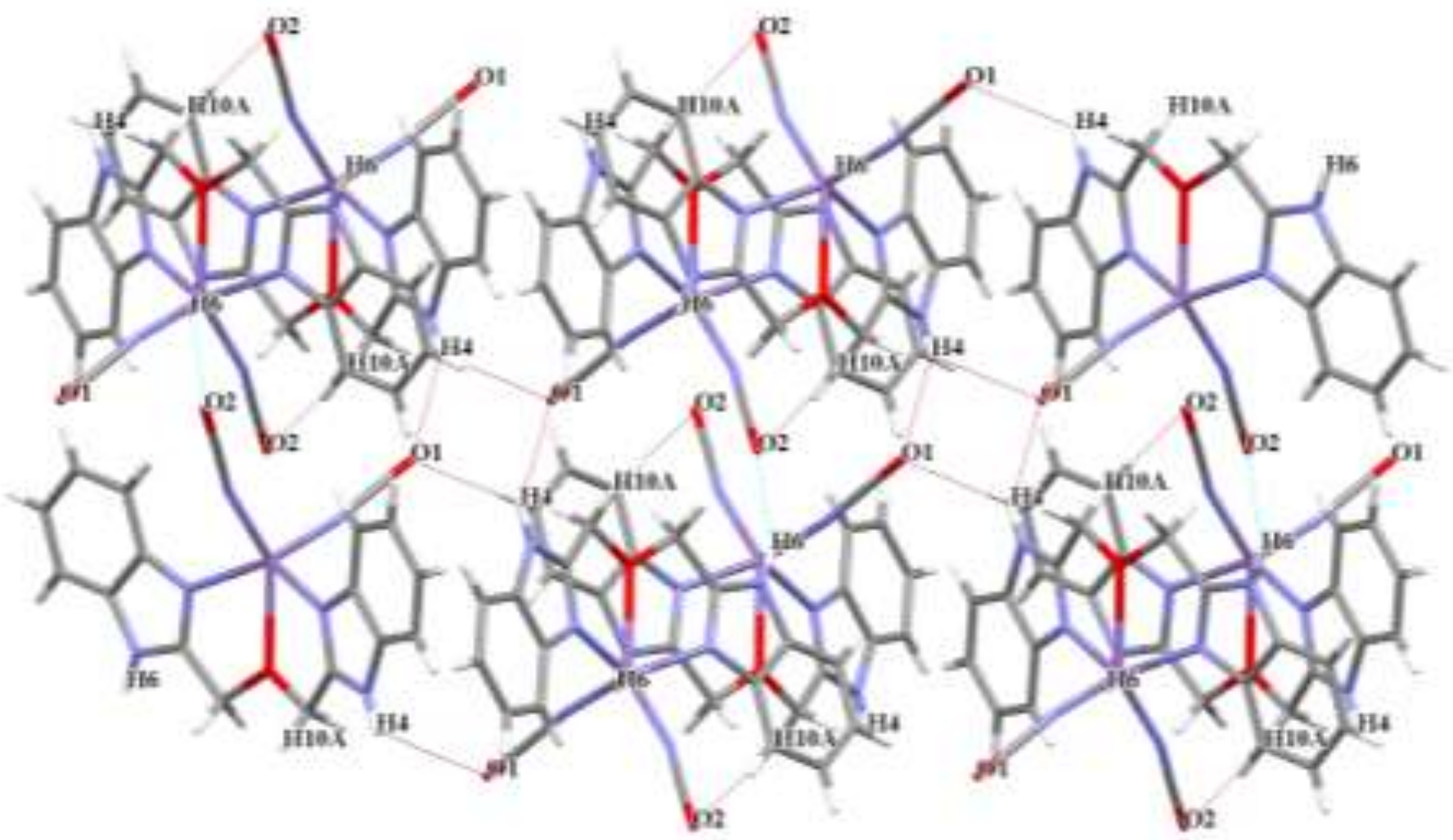

Figure S6. Crystal packing of **(2)** showing the formation of the hydrogen bonds between the hydrogens N–H of the benzimidazole rings and oxygen atoms of the cyanate ions, along with C(10)–H(10A)•••O(2) short contacts. The detailed characteristics of these contacts are given in **Table S4**.

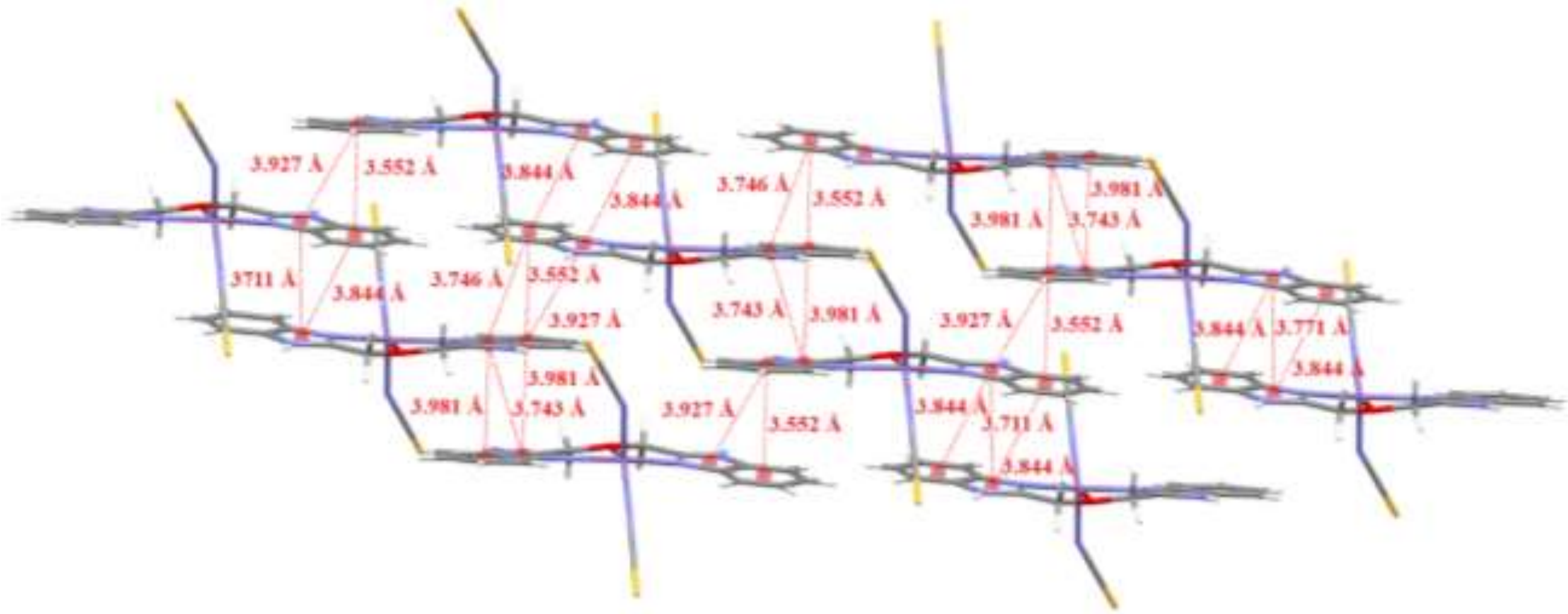

Figure S7. Crystal packing of **(1)** showing π•••π interactions with Cg(I)•••Cg(J) distances below 4 Å. The detailed characteristics of these contacts are given in **Table S5**.

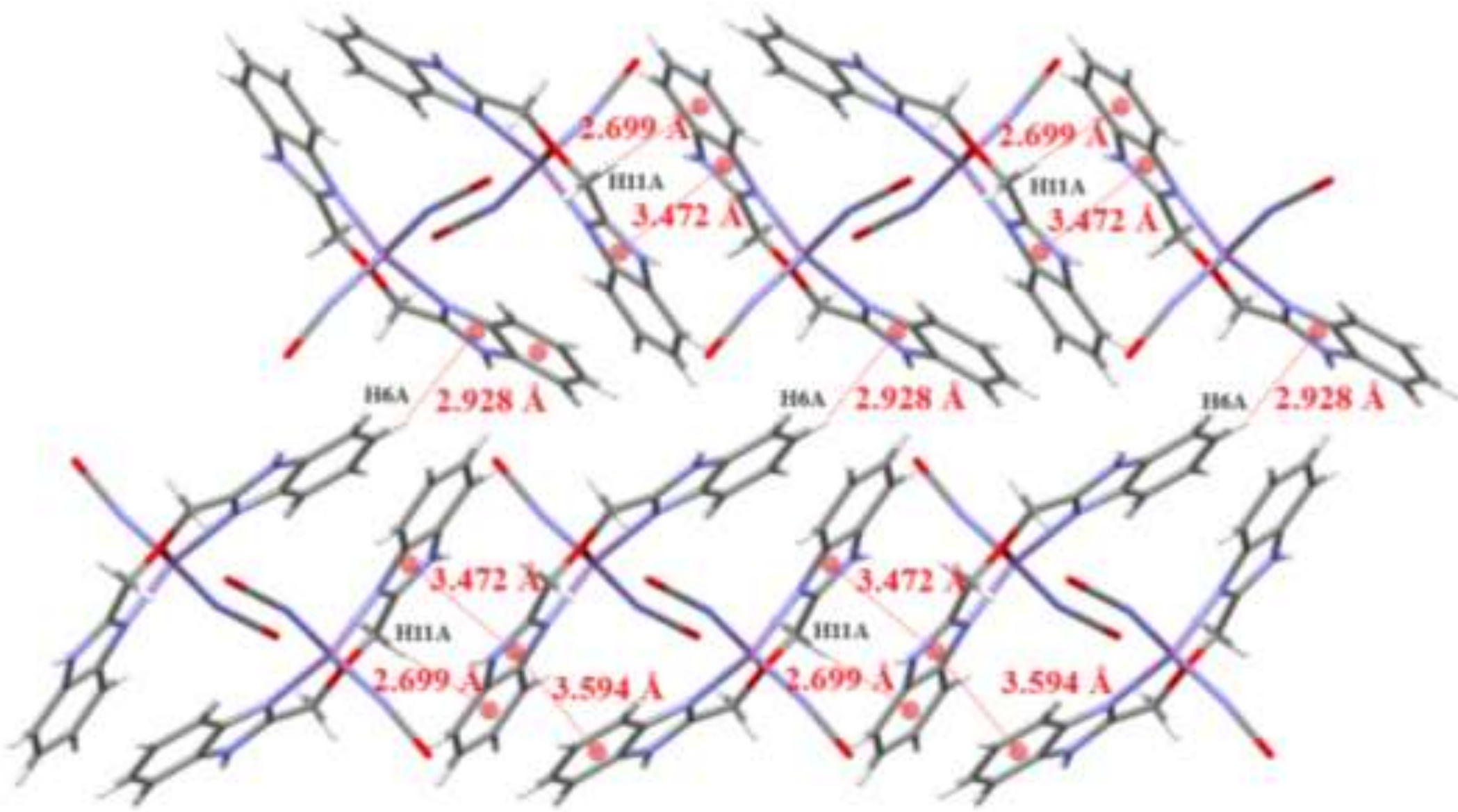

Figure S8. Crystal packing of **(2)** showing π•••π interactions with Cg(I)•••Cg(J) distances below 4 Å along with X—H•••Cg(J)(π-ring) interactions. The detailed characteristics of these contacts are given in **Tables S5 and S6**.

5. Theoretical calculations

Geometry optimization performed using the procedure described in experimental section (main text) revealed that the main structural changes in both complexes involve the Mn–O interaction, while the Mn–N coordination framework remains relatively rigid. In complex **(1)**, the Mn–O bond length increased from 2.382 to 2.566 Å, whereas in complex **(2)** a larger elongation from 2.349 to 2.701 Å was observed, indicating weaker Mn–O interaction in **(2)**. In contrast, Mn–N bond lengths and most

angular parameters changed only slightly during optimization. The largest angular variations involved angles containing the donor oxygen atom, suggesting that structural relaxation occurs mainly along the Mn–O axis. In both systems, the optimized geometries retained a distorted square-pyramidal coordination environment around the Mn(II) center. The calculations confirmed preservation of the high-spin electronic state and showed stable convergence behavior, indicating reliable description of the electronic structure.

Table S8. Selected theoretically calculated bond lengths [Å] and angles [°] of **(1)** and **(2).**

**(1)**			**(2)**		
	**exp**	**theor.**		**exp.**	**theor.**
Mn(1)-O(1)	2,38	2,56	Mn(1)–O(3)	2,35	2,70
Mn(1)–N(3)	2,15	2,29	Mn(1)–N(1)	2,06	2,09
Mn(1)–N(5)	2,16	2,29	Mn(1)–N(2)	2,04	2,05
Mn(1)–N(1)	2,08	2,14	Mn(1)–N(3)	2,19	2,33
Mn(1)–N(2)	2,08	2,08	Mn(1)–N(5)	2,18	2,33
N(1)–Mn(1)–N(2)	109,09	128,14	N(1)–Mn(1)–N(2)	110,83	132,62
N(1)–Mn(1)–N(3)	95,46	105,42	N(1)–Mn(1)–N(3)	107,90	96,67
N(1)–Mn(1)–N(5)	110,26	105,38	N(1)–Mn(1)–N(5)	105,81	96,58
N(2)–Mn(1)–N(3)	102,30	96,38	N(2)–Mn(1)–N(3)	99,08	105,32
N(2)–Mn(1)–N(5)	101,19	96,39	N(2)–Mn(1)–N(5)	99,46	105,13
N(3)–Mn(1)–N(5)	136,80	127,88	N(3)–Mn(1)–N(5)	132,23	123,01
N(1)–Mn(1)–O(1)	113,82	147,90	N(1)–Mn(1)–O(3)	107,90	81,60
N(2)–Mn(1)–O(1)	136,702	83,94	N(2)–Mn(1)–O(3)	141,26	145,80
N(3)–Mn(1)–O(1)	68,64	65,72	N(3)–Mn(1)–O(3)	69,00	63,80
N(5)–Mn(1)–O(1)	69,35	65,75	N(5)–Mn(1)–O(3)	69,03	63,80

6. Magnetic data

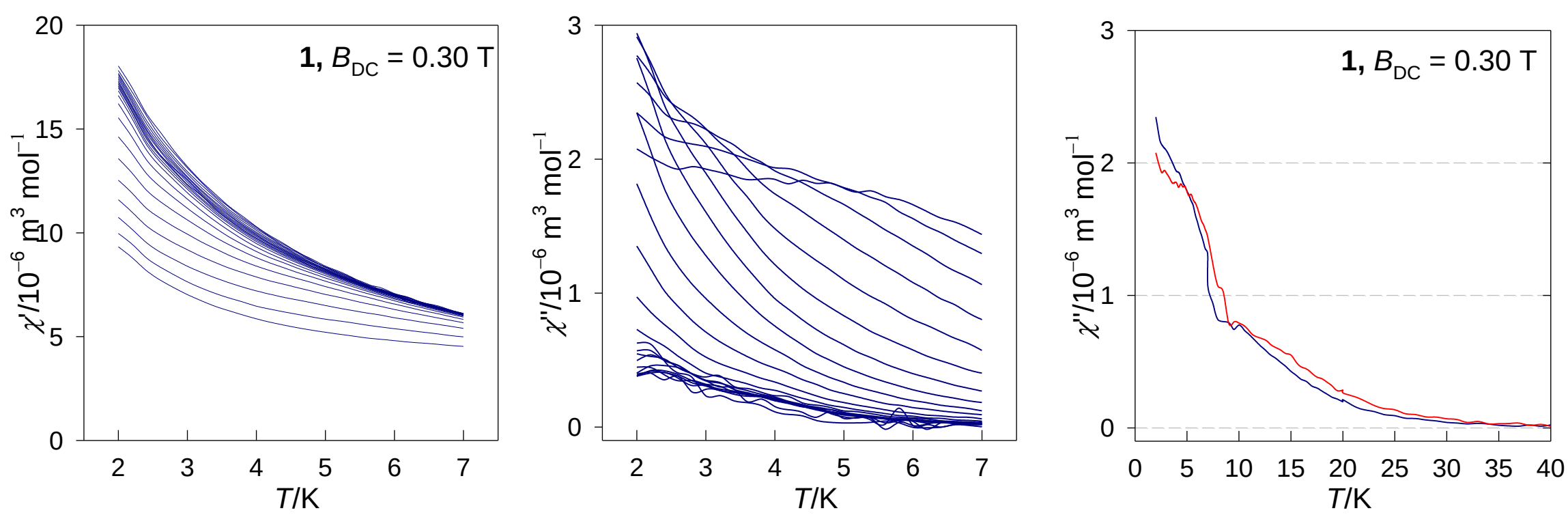

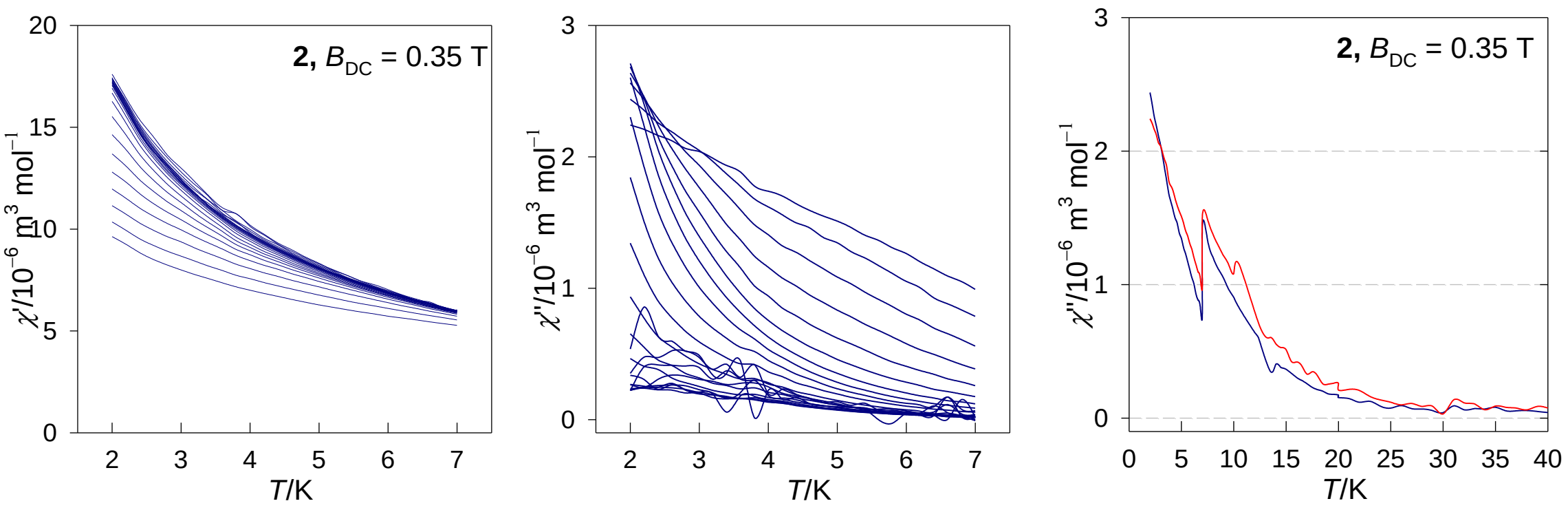

Figure S9. Temperature evolution of the AC susceptibility for varied frequencies.

Table S9. Temperature dependence of AC susceptibility parameters for **(1)** at B_{DC} = 0.30 T.

T/K	$R(\chi')$ /%	$R(\chi'')$ /%	χ_S	χ_{LF}	α_{LF}	τ_{LF} / s	χ_{HF}	α_{HF}	τ_{HF} /10^{-3} s	x_{LF}	x_{HF}
2.0	0.59	5.1	7.6(1)	9.1(6)	.13	2.0(11)	19.0(5)	.30	0.61(2)	.13	.87
2.2	0.52	4.5	7.1(1)	8.7(5)	.22	1.7(8)	17.9(4)	.30	0.54(2)	.15	.85
2.4	0.67	3.9	6.4(1)	7.4(3)	.10	0.77(16)	16.0(2)	.32	0.47(1)	.11	.89
2.6	0.72	3.9	5.5(2)	6.5(3)	.10	0.74(16)	15.1(2)	.34	0.38(2)	.11	.89
2.8	0.33	2.6	5.4(8)	6.4(2)	.16	0.81(12)	14.3(1)	.31	0.36(1)	.11	.89
3.0	0.33	3.3	5.3(1)	6.5(3)	.40	1.0(5)	13.7(2)	.28	0.33(1)	.13	.87
3.4	0.36	2.5	4.7(1)	5.6(2)	.30	0.76(18)	12.3(1)	.28	0.26(1)	.13	.87
4.0	0.25	1.0	3.5(1)	4.0(1)	.23	0.34(3)	10.4(1)	.30	0.17(1)	.08	.92
4.6	0.37	3.0	3.5(1)	4.0(1)	.30	0.33(8)	9.2(1)	.21	0.16(1)	.09	.91
5.2	0.21	1.7	3.3(1)	3.5(1)	.30	0.19(3)	8.1(1)	.17	0.14(1)	.06	.94
5.8	0.28	3.3	3.0(1)	3.2(1)	.30	0.68(38)	7.4(1)	.15	0.12(1)	.05	.95
6.4	0.10	1.4	2.7(1)	2.8(1)	.30	0.14(3)	6.7(1)	.14	0.10(1)	.02	.98
7.0	0.25	2.1	2.6(1)	2.7(1)	.30	0.16	6.2(1)	.13	0.092(3)	.03	.97

[a] Obtained by a two-set Debye model; χ in units of 10^{-6} m^3 mol^{-1}. $R(\chi')$ and $R(\chi'')$ – discrepancy factors of the fit. Standard deviation of the last digit in parentheses.

Table S10. Temperature dependence of AC susceptibility parameters for **(2)** at B_{DC} = 0.35 T.[a]

T/K	$R(\chi')$ /%	$R(\chi'')$ /%	χ_S	χ_{LF}	α_{LF}	τ_{LF} /s	χ_{IF}	α_{IF}	τ_{IF} /10^{-3} s	χ_{HF}	α_{HF}	τ_{HF} /10^{-3} s	x_{LF}	x_{HF}
2.0	0.18	1.6	7.0(2)	9.8(23)	.18	10.0	12.8(28)	.03	1.8(1)	20.0(22)	.32	0.20(3)	.21	.56
2.2	0.25	2.1	6.4(3)	9.9(22)	.13	7.6	12.5(29)	.05	1.6(2)	19.5(20)	.32	0.17(4)	.27	.54
2.4	0.30	1.9	5.6(9)	9.8(49)	.32	16.3	12.6(67)	.13	1.3(3)	19.0(42)	.32	0.10(4)	.31	.48
2.6	0.31	1.3	5.5(6)	9.9(45)	.38	18.6	11.6(56)	.09	1.5(4)	18.3(40)	.32	0.13(4)	.35	.52
2.8	0.29	2.1	5.5(6)	9.9(37)	.37	19.9	11.7(40)	.14	1.4(5)	17.5(35)	.26	0.12(2)	.37	.48
3.0	0.32	2.0	5.7(4)	9.8(34)	.41	19.9	11.6(32)	.13	1.3(3)	16.4(34)	.16	0.12(1)	.39	.45
3.4	0.35	2.2	4.8(4)	5.6(5)	.04	1.1	6.3(8)	.07	2.5(7)	12.0(1)	.26	0.11(1)	.12	.79
4.0	0.24	2.9	4.7(3)	5.6(3)	.37	1.5	6.5(5)	.19	1.9(6)	10.6(2)	.13	0.093(5)	.16	.70
4.6	0.35	4.1	4.7(3)	5.6(5)	.49	2.0	6.5(9)	.22	0.81	9.5(5)	.01	0.090(7)	.19	.62
5.2	0.27	2.9	4.5(2)	5.1(4)	.49	2.0	5.6(6)	.23	0.81	8.3(3)	.01	0.094(4)	.14	.71
5.8	0.27	3.3	4.4(1)	4.7(4)	.37	2.0	4.9(4)	.28	1.6	7.4(4)	.01	0.094(4)	.10	.81
6.4	0.20	3.6	3.9(1)	4.0(1)	.10	1.3	4.1(2)	.20	2.0	4.4(22)	.04	0.075(5)	.05	.90
7.0	0.35	3.7	3.9(1)	4.0(4)	.49	2.0	4.1(3)	.09	4.0	6.1(3)	.01	0.076(6)	.06	.91

[a] Mole fractions $x_{LF} + x_{IF} + x_{HF} = 1$.

7. EPR spectra

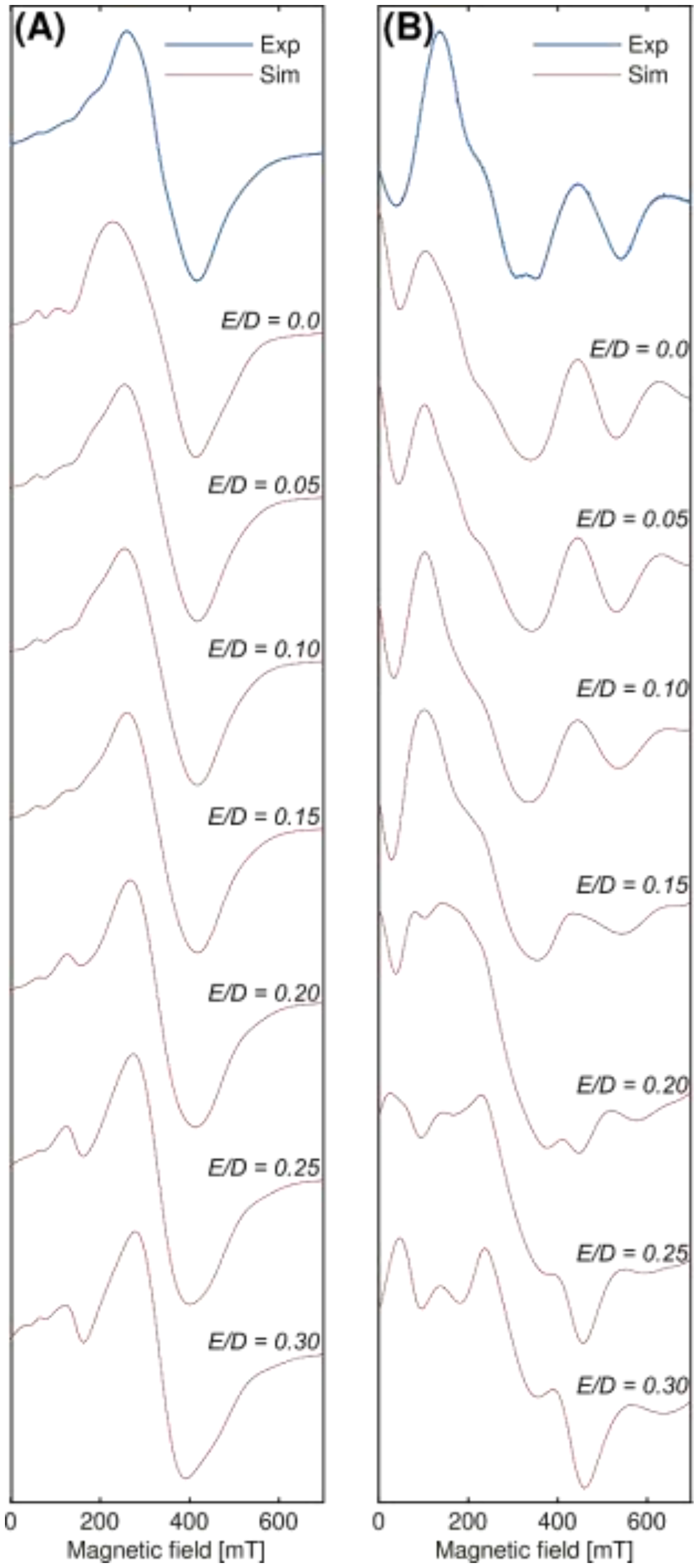

Figure S10. Dependence of the simulated X-band EPR spectra of complexes **1** (A) and **2** (B) on the rhombicity parameter *E/D*, assuming fixed values of |D| = 0.06 cm^{-1} for **1** and |D|=0.14 cm^{-1} for **2**. For complex **1**, the best agreement with the experimental spectrum is obtained for E/D values between 0.05 and 0.15, whereas for complex **2** the optimum fit is found at E/D=0.10.

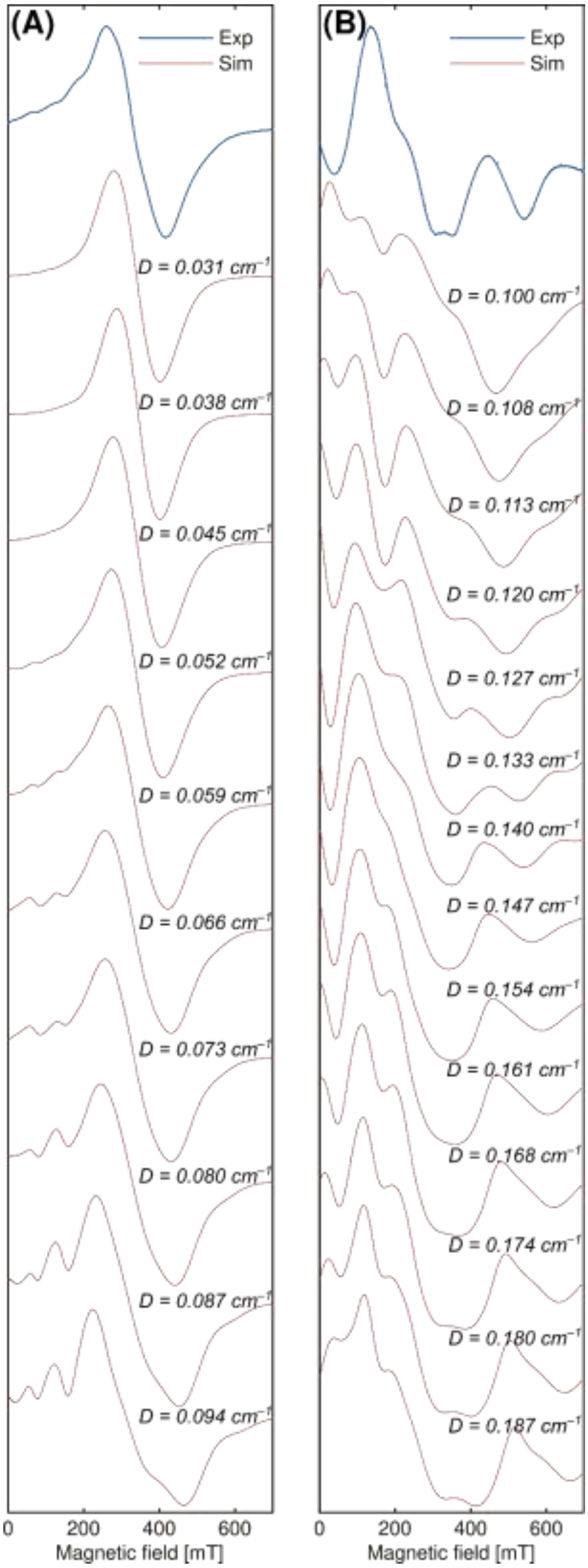

Figure S11. Dependence of the simulated X-band EPR spectra of complexes **1** (A) and **2** (B) on the axial parameter |*D*|, assuming fixed values of *E/D* = 0.0072 for **1** and *E/D* = 0.14 for **2**. For complex **1**, the best agreement with the experimental spectrum is obtained for |*D*| = 0.059 cm^{-1}, whereas for complex **2** the optimum fit is found at D=0.14 cm^{-1}.

8. References

[1] Matthews, C. J.; Leese, T. A.; Clegg, W.; Elsegood, M. R. J.; Horsburgh, L.; Lockhart, J. C. A Route to Bis(benzimidazole) Ligands with Built-In Asymmetry: Potential Models of Protein Binding Sites Having Histidines of Different Basicity. Inorg. Chem.1996, 35, 7563-7571.

[2] Llunell, M.; Casanova, D.; Cirera, J.; Alemany, P.; Gómez-Ruiz, S. SHAPE version 2.0., Universitat de Barcelona, 2010.

[3] Addison, A. W.; Rao T. N.; Reedijk, J.; van Rijn, J.; and Verschoor, G. C. Synthesis, structure, and spectroscopic properties of copper(II) compounds containing nitrogen–sulphur donor ligands; the crystal and molecular structure of aqua[1,7-bis(N-methylbenzimidazol-2′-yl)-2,6-dithiaheptane]copper(II) perchlorate. J. Chem. Soc., Dalton Trans., 1984, 1349-1356.

[4] Adhikary, J.; Chakraborty, A.; Dasgupta, S.; Chattopadhyay, S. K.; Kruszynski, R.; Trzesowska-Kruszynska, A.; Stepanović, S.; Gruden-Pavlović, M.; Swart M.; and Das, D. Unique mononuclear Mn^{II} complexes of an end-off compartmental Schiff base ligand: experimental and theoretical studies on their bio-relevant catalytic promiscuity. Dalton Trans., 2016, 45, 12409–12422.

[5] Kose, M.; McKee, V. Mn(II) complexes of a tridentate benzimidazole ligand: Synthesis, structural characterization and catalase mimetic studies. Polyhedron 2014, 75, 30–39.

[6] Yu, L.; Shi, J.-M.; Zhang Y.-Q.; Wang, Y.-Q.; Fan, Y.-N.; Zhang, G.-Q.; Shi, W.; Cheng, P. π- π Stacking and magnetic coupling mechanism on a mono-nuclear Mn(II) complex. J. Mol. Struct. 2011, 987, 138–143.

[7] Mantel, C.; Baffert, C.; Romero, I.; Deronzier, A.; Pécaut, J.; Collomb M-N.; and Duboc, C. Structural Characterization and Electronic Properties Determination by High-Field and High Frequency EPR of a Series of Five-Coordinated Mn(II) Complexes. Inorg. Chem. 2004, 43, 6455-6463.

[8] Groom, C. R.; Bruno, I. J.; Lightfoot M. P.; and Ward, S. C. The Cambridge Structural Database. Acta Cryst. 2016. B72, 171-179.

[9] Machura, B.; Palion, J.; Mroziński, J.; Kalińska, B.; Amini, M.; Najafpour, M. M.; Kruszynski, R. Manganese(II) complexes of 2,3,5,6-tetra-(2-pyridyl)pyrazine – Syntheses, crystal structures, spectroscopic, magnetic and catalytic propetries. Polyhedron, 2013, 53, 132–143.

[10] Savard, D.; and Leznoff, D. B. Synthesis, structure and light scattering properties of tetraalkylammonium metal isothiocyanate salts. Dalton Trans., 2013, 42, 14982–14991.

[11] Neumann, T.; Jess I.; and Näther C. Synthesis and Magnetic Properties of Solid Solutions of Ferromagnetic Layered Coordination Polymers Prepared from Solution and the Solid State. Acta Crystallogr., Sect. E: Crystallogr. Commun., 2018, 74, 15–20.
